# Inhibition of UBE2C Promotes Parkin‐Mediated K63‐Linked Ubiquitination of TOP2A to Induce Senescence and Increase Sensitivity of Doxorubicin in Breast Cancer

**DOI:** 10.1002/advs.202417348

**Published:** 2025-07-29

**Authors:** Yihui Yang, Wan Li, Hong Yang, Fang Xu, Sen Zhang, Wanxin Cao, Xiaoxue Li, Xu Zhang, Xiangyin Chi, Hongquan Wang, Guanhua Du, Yumin Wang, Jinhua Wang

**Affiliations:** ^1^ The State Key Laboratory of Bioactive Substance and Function of Natural Medicines Beijing 100050 China; ^2^ Beijing Key Laboratory of Innovative Drug Discovery and Polymorphic Druggability Research for Cerebrovascular Diseases, Institute of Materia Medica Chinese Academy of Medical Science and Peking Union Medical College Beijing 100050 China; ^3^ Department of Geriatrics, Aerospace Center Hospital Peking University Aerospace School of Clinical Medicine Beijing 100049 China; ^4^ Department of Respiratory and Critical Care Medicine, Aerospace Center Hospital Peking University Aerospace School of Clinical Medicine Beijing 100049 China

**Keywords:** breast cancer, doxorubicin sensitivity, senescence, TOP2A, UBE2C

## Abstract

Breast cancer is now the second most commonly diagnosed cancer and the most common female malignancy. Chemotherapy‐based adjuvant therapy after surgery and neoadjuvant therapy before surgery are cornerstones of breast cancer treatment. Doxorubicin is one of the most commonly used anthracycline chemotherapy treatments for breast cancer; however, doxorubicin resistance is a major barrier to its clinical use. Therefore, there is an urgent need to discover new targets to overcome doxorubicin resistance in breast cancer. Ubiquitin‐conjugating enzyme 2C (UBE2C) is an E2 ubiquitin‐conjugating enzyme that catalyzes the assembly of K11‐linked ubiquitin chains. In recent years, dysregulation of UBE2C has been implicated in a variety of cancers, including breast cancer; however, the underlying mechanisms remain unclear. In the present study, UBE2C was found to be markedly upregulated in breast cancer and transcriptionally regulated by FOXM1. Inhibition of UBE2C suppressed proliferation and induced senescence in breast cancer cells. Moreover, the inhibition of UBE2C promoted Parkin‐mediated K63‐linked ubiquitination of TOP2A, leading to its proteasomal degradation and thus sensitizing breast cancer cells to doxorubicin. The study reveals that UBE2C is a critical regulator of breast cancer cell proliferation, senescence, and sensitivity to doxorubicin.

## Graphical abstract

In the study, UBE2C was found to be significantly overexpressed in breast cancer and transcriptionally regulated by FOXM1. Inhibition of UBE2C suppressed proliferation, induced senescence, and sensitized breast cancer cells to doxorubicin. Mechanistically, UBE2C inhibition promoted Parkin‐mediated K63‐linked ubiquitination of TOP2A. The study reveals that UBE2C is a critical regulator of breast cancer cell proliferation, senescence, and sensitivity to doxorubicin.

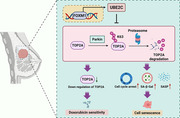

## 1 Introduction

Breast cancer is now the second diagnosed and the most common female malignancy worldwide, with an estimated 2.3 million new cases, according to GLOBOCAN 2022.^[^ ^1^ ^]^ Based on the expression status of estrogen receptor (ER), progesterone receptor (PgR) and human epidermal growthFactor receptor 2, (HER2), breast cancer can be classified into three subtypes: luminal‐like, HER2‐positive, and triple negative breast cancer (TNBC).^[^ ^2^ ^]^ Chemotherapy‐based adjuvant therapy after surgery and neoadjuvant therapy before surgery have become the cornerstones of breast cancer treatment.^[^ ^3^ ^]^ Anthracycline and taxane‐based chemotherapy can substantially improve the outcomes of breast cancer, reducing its recurrence and mortality.^[^ ^4^ ^]^ Anthracyclines are the most effective and common chemotherapy treatments for breast cancer, among which doxorubicin is one of the most commonly used in clinical practice.^[^ ^5^ ^]^

Anthracycline doxorubicin is a widely used chemotherapeutic agent targeting topoisomerase II and has been used to treat a plethora of cancers including breast cancer, lung cancer, bladder cancer.^[^ ^6^, ^7^ ^]^ The anti‐cancer mechanisms of doxorubicin mainly involve topoisomerase II inhibition, DNA intercalation, free radical generation, and induction of oxidative stress.^[^ ^8^ ^]^ Doxorubicin induces cell cycle arrest and triggers apoptosis to inhibit proliferation of cancer cell.^[^ ^9^ ^]^ Ferroptosis is another type of programmed cell death induced by doxorubicin and it is associated with doxorubicin cardiotoxicity.^[^ ^10^ ^]^ Like most other chemotherapeutic agents, doxorubicin induces senescence in a wide range of cancer cells, a phenomenon called therapy‐induced senescence (TIS).^[^ ^11^ ^]^ Triggering cellular senescence using chemotherapeutic agents is a novel cancer treatment approach. Doxorubicin induced‐senescence reshapes the breast‐brain metastasis microenvironment and enhances the efficacy of anti‐PD1 therapy.^[^ ^12^ ^]^ However, doxorubicin resistance is a great barrier to treatment of breast cancer, and several potential mechanisms have been proposed, including alterations or mutations in topoisomerase II,^[^ ^13^ ^]^ overexpression of ATP‐binding cassette (ABC) transporters,^[^ ^14^ ^]^ dysregulation of proliferation and DNA repair signaling pathways,^[^ ^15^ ^]^ autophagy alterations,^[^ ^16^ ^]^ and increased cancer cells stemness.^[^ ^17^ ^]^ To overcome doxorubicin resistance, doxorubicin and siRNA were incorporated into nanoparticles to simultaneously promote doxorubicin accumulation and inhibit oncogenic pathways in cancer cells.^[^ ^18^ ^]^ However, their application in clinical treatment requires further validation.

Ubiquitin‐conjugating enzyme 2C (UBE2C) is an E2 ubiquitin‐conjugating enzyme that catalyzes the assembly of K11‐linked ubiquitin on target proteins.^[^ ^19^ ^]^ By interacting with the anaphase‐promoting complex/cyclosome (APC/C), UBE2C mediates the ubiquitination and degradation of securin and cyclin B to promote mitotic exit and G1/S transition.^[^ ^20^ ^]^ In recent years, several studies have demonstrated that dysregulation of UBE2C is implicated in various cancers including breast, lung, endometrial, cervical, ovarian, pancreatic, and gastric cancers.^[^ ^21^, ^22^, ^23^, ^24^, ^25^, ^26^ ^]^ In lung cancer, UBE2C facilitates tumorigenesis by interacting with CDH1 to promote the ubiquitin and degradation of the DEP domain–containing mechanistic target of rapamycin (mTOR) interacting protein (DEPTOR), thus activating mTOR signaling.^[^ ^21^ ^]^ UBE2C also inhibits autophagy and contributes to endometrial cancer progression by inducing K48‐linked ubiquitination and degradation of sirtuin 1 (SIRT1).^[^ ^22^ ^]^ In breast cancer, overexpression of UBE2C is associated with a worse prognosis, and inhibition of UBE2C can suppress the proliferation and invasion of breast cancer cells.^[^ ^26^, ^27^, ^28^ ^]^ However, the mechanisms of by which UBE2C drives the tumorigenesis of breast cancer remain unclear.

In this study, we showed that UBE2C inhibition suppressed the proliferation of breast cancer cells and induced senescence. This inhibition also downregulated the expression of topoisomerase II‐ alpha (TOP2A), sensitizing breast cancer cells to doxorubicin and aggravating doxorubicin‐induced senescence. Mechanistically, UBE2C inhibition promoted Parkin‐mediated K63‐linked ubiquitination of TOP2A and led to its proteasomal degradation. Overexpression of TOP2A reversed the cell senescence induced by UBE2C inhibition. Moreover, aberrant UBE2C expression was found to be transcriptionally regulated by forkhead box protein M1 (FOXM1) in breast cancer. In vivo, UBE2C inhibition also sensitized breast cancer cells to doxorubicin. All in all, UBE2C inhibition could promote Parkin‐mediated K63‐linked ubiquitination of TOP2A to induce senescence and increase doxorubicin sensitivity in breast cancer.

## 2 Results

### 2.1 UBE2C is Upregulated in Breast Cancer and Correlated with Poor Prognosis

**Figure 1:**
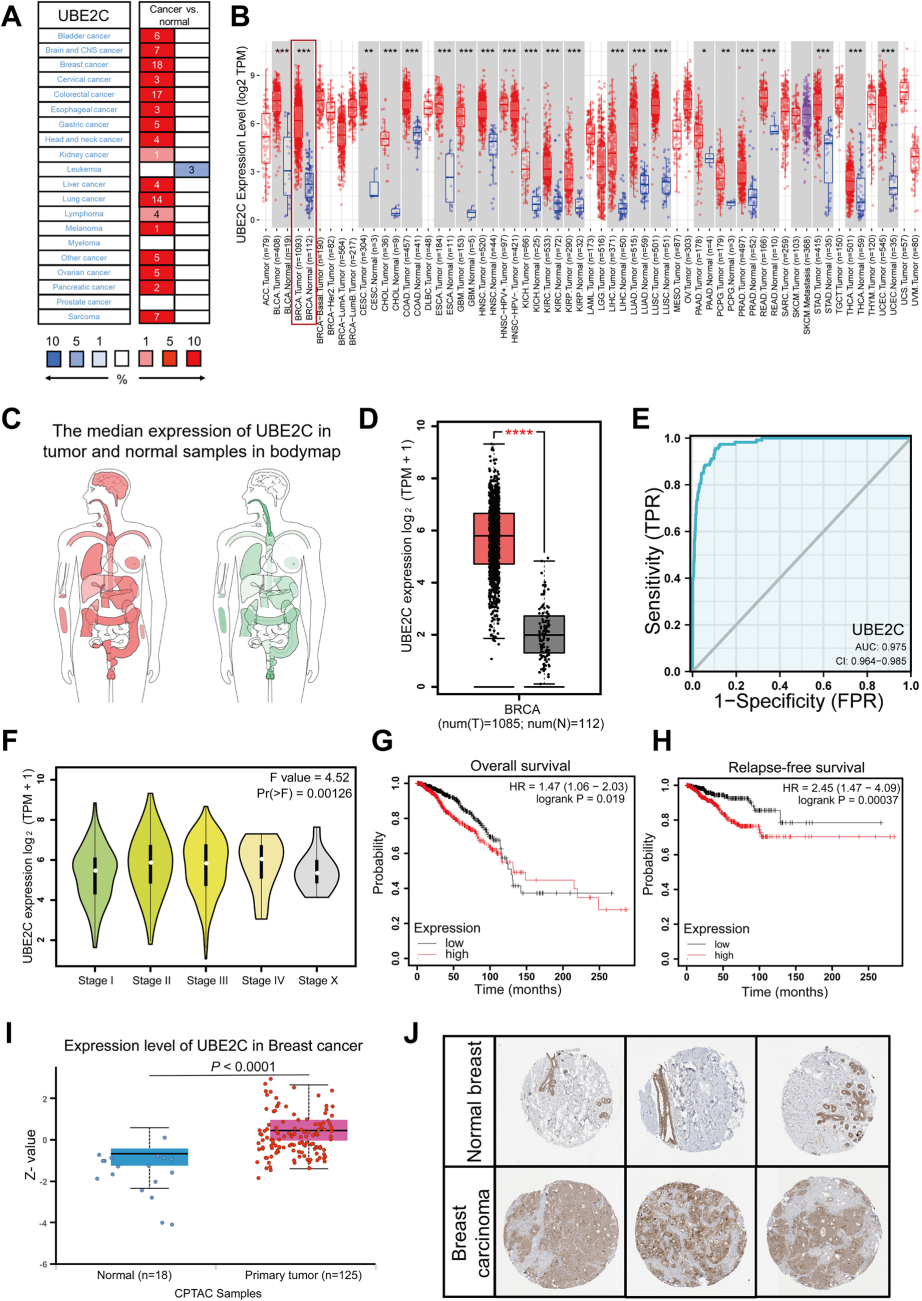
UBE2C is upregulated in breast cancer and correlated with poor prognosis. A) The Oncomine database was used to analyze the expression of UBE2C in pan‐cancers. There are 109 datasets were involved in the analysis and UBE2C was overexpressed in 106 datasets. B) TIMER database was used to analyze the expression of UBE2C in pan‐cancers. ACC.Tumor (n = 79); BLCA.Tumor (n = 408); BLCA.Normal (n = 19); BRCA.Tumor (n = 1093); BRCA.Normal (n = 112); CESC.Tumor (n = 304); CESC.Normal (n = 3); CHOL.Tumor (n = 36); CHOL.Normal (n = 9); COAD.Tumor (n = 457); COAD.Normal (n = 41); DLBC.Tumor (n = 184); ESCA.Tumor (n = 184); ESCA.Normal (n = 11); GBM.Tumor (n = 153); GBM.Normal (n = 5); HNSC.Tumor (n = 520); HNSC.Normal (n = 44); KICH.Tumor (n = 66); KICH.Normal (n = 25); KIRC.Tumor (n = 533); KIRC.Normal (n = 72); KIRP.Tumor (n = 290); KIRP.Normal (n = 32); LAML.Tumor (n = 173); LGG.Tumor (n = 516); LIHC.Tumor (n = 371); LIHC.Normal (n = 50); LUAD.Tumor (n = 515); LUAD.Normal (n = 59); LUSC.Tumor (n = 501); LUSC.Normal (n = 51); MESO.Tumor (n = 87); OV.Tumor (n = 303); PAAD.Tumor (n = 178); PAAD.Normal (n = 4); PCPG.Tumor (n = 179); PCPG.Normal (n = 3); PRAD.Tumor (n = 497); PRAD.Normal (n = 52); READ.Tumor (n = 166); READ.Normal (n = 10); SARC.Tumor (N = 259); SKCM.Tumor (n = 103); STAD.Tumor (n = 415); STAD.Normal (n = 35); TGCT.Tumor (n = 150); THCA.Tumor (n = 501); THCA.Normal (n = 59); THYM.Tumor (n = 120); UCEC.Tumor (n = 545); UCEC.Normal (n = 35); UCS.Tumor (n = 57); UVM.Tumor (n = 80); C) GEPIA database was used to depict the average expression of UBE2C in tumor and normal samples in bodymap. D) GEPIA database was used to analyze the expression of UBE2C in breast cancer. Tumor (n = 1085); Normal (n = 112). E) ROC plot was used to depict the potential of UBE2C as a diagnostic marker for breast cancer using the data from TCGA breast cancer cohorts. Tumor (n = 1085); Normal (n = 112). F) GEPIA database was used to analyze the expression of UBE2C in different stages of breast cancer and normal breast tissues based on the data from TCGA breast cancer cohorts. Tumor (n = 1085); Normal (n = 112). G) Kaplan–Meier plotter database was used to analyze the correlation of the expression of UBE2C with the OS of breast cancer patients based on Breast Cancer RNAseq dataset (n = 1538). H) Kaplan–Meier plotter database was used to analyze the correlation of the expression of UBE2C with the RFS of breast cancer patients based on Breast Cancer RNAseq dataset (n = 1291). I) CPTAC database was used to analyze the protein level of UBE2C in breast cancer samples and normal breast samples. Tumor (n = 125); Normal (n = 18). J) The representative images of the expression of UBE2C in normal breast tissues and breast cancer tissues were obtained from HPA database and displayed. *P < 0.05, **P < 0.01, ***P < 0.001, ****P < 0.0001 versus normal tissue group.

To explore the pan‐cancer expression of UBE2C, the Oncomine and TIMER databases were used to analyze the mRNA levels of UBE2C across all tumor types in the cancer genome atlas (TCGA). UBE2C was significantly upregulated in almost all cancers types including breast cancer (**Figure** **1**A,B). The expression of UBE2C in breast cancer was also validated using several the gene expression omnibus (GEO) datasets (Figure S1A, Supporting Information) and TCGA database (Figure 1C,D). To further validate the diagnostic role of UBE2C in breast cancer, receiver operating characteristic (ROC) curve analysis was used to differentiate tumor samples from normal tissues (Figure 1E). UBE2C showed a great potential as a diagnostic marker for breast cancer, with an AUC of 0.975. Considering that there are three major molecular subtypes of breast cancer, the mRNA levels of UBE2C in luminal‐like, HER2 positive, and TNBC were analyzed based on TCGA database (Figure S1B, Supporting Information).^[^ ^2^ ^]^ The expression of UBE2C was significantly upregulated in all subtypes of breast cancer compared to normal breast tissue, and TNBC harbored the highest expression level of UBE2C among all subtypes. Moreover, the expression of UBE2C was upregulated at higher pathological stages and histological grades of breast cancer (Figure 1F; Figure S1C,D, Supporting Information). To further confirm the prognostic value of UBE2C in breast cancer, the Kaplan–Meier plotter database was used to analyze the association between UBE2C expression and survival outcomes in patients with breast cancer. Higher expression of UBE2C was correlated with worse prognosis in breast cancer with regards to overall survival (OS) (Figure 1G) and relapse‐free survival (RFS) (Figure 1H). Moreover, UBE2C expression correlated with poor prognosis in multiple GEO datasets (**Table** **1**). The protein level of UBE2C in breast cancer was also significantly upregulated based on the the national cancer institute'sclinical proteomic tumor analysis consortium (CPTAC) and the Human Protein Atlas databases (Figure 1I,J). Taken together, these results indicated that UBE2C was upregulated in breast cancer and correlated with poor prognosis in patients with breast cancer.

**Table 1:** The prognosis significance of UBE2C in breast cancer based on multiple GEO datasets.

DATASET	ENDPOINT	N	COX *P*‐VALUE	ln(HR_high_ / HR_low_)	ln(HR)	HR [95% CI^low^ – CI^upp^]
GSE3143	Overall Survival	158	0.001682	0.99	0.65	1.92 [1.28 – 2.89]
GSE12276	Relapse Free Survival	204	0.048484	0.48	0.19	1.21 [1.00 – 1.46]
GSE11121	Distant Metastasis Free Survival	200	0.000189	1.18	0.82	2.28 [1.48 – 3.51]
GSE9893	Overall Survival	155	0.000612	1.39	0.44	1.55 [1.21 – 2.00]
GSE2034	Distant Metastasis Free Survival	286	0.000613	0.7	0.62	1.85 [1.30 – 2.64]
GSE1456‐GPL96	Overall Survival	159	0.001118	1.58	0.79	2.21 [1.37 – 3.55]
GSE1456‐GPL96	Relapse Free Survival	159	0.0005	1.42	0.84	2.30 [1.44 – 3.69]
GSE1456‐GPL96	Disease Specific Survival	159	0.000456	1.55	1	2.71 [1.55 – 4.75]
GSE7378	Disease Free Survival	54	0.010747	2.53	1.05	2.85 [1.27 – 6.38]
GSE3494‐GPL96	Disease Specific Survival	236	0.000108	1.15	0.86	2.37 [1.53 – 3.66]
GSE4922‐GPL96	Disease Free Survival	249	0.00024	0.99	0.66	1.94 [1.36 – 2.75]
GSE2990	Relapse Free Survival	62	0.017916	1.82	0.58	1.79 [1.11 – 2.90]
GSE2990	Distant Metastasis Free Survival	54	0.004891	2.43	0.9	2.45 [1.31 – 4.58]
GSE7390	Overall Survival	198	0.021472	0.96	0.31	1.37 [1.05 – 1.78]
GSE7390	Distant Metastasis Free Survival	198	0.038837	0.8	0.27	1.31 [1.01 – 1.70]

### 2.2 Inhibition of UBE2C Suppresses Proliferation and Induces Senescence in Breast Cancer

**Figure 2:**
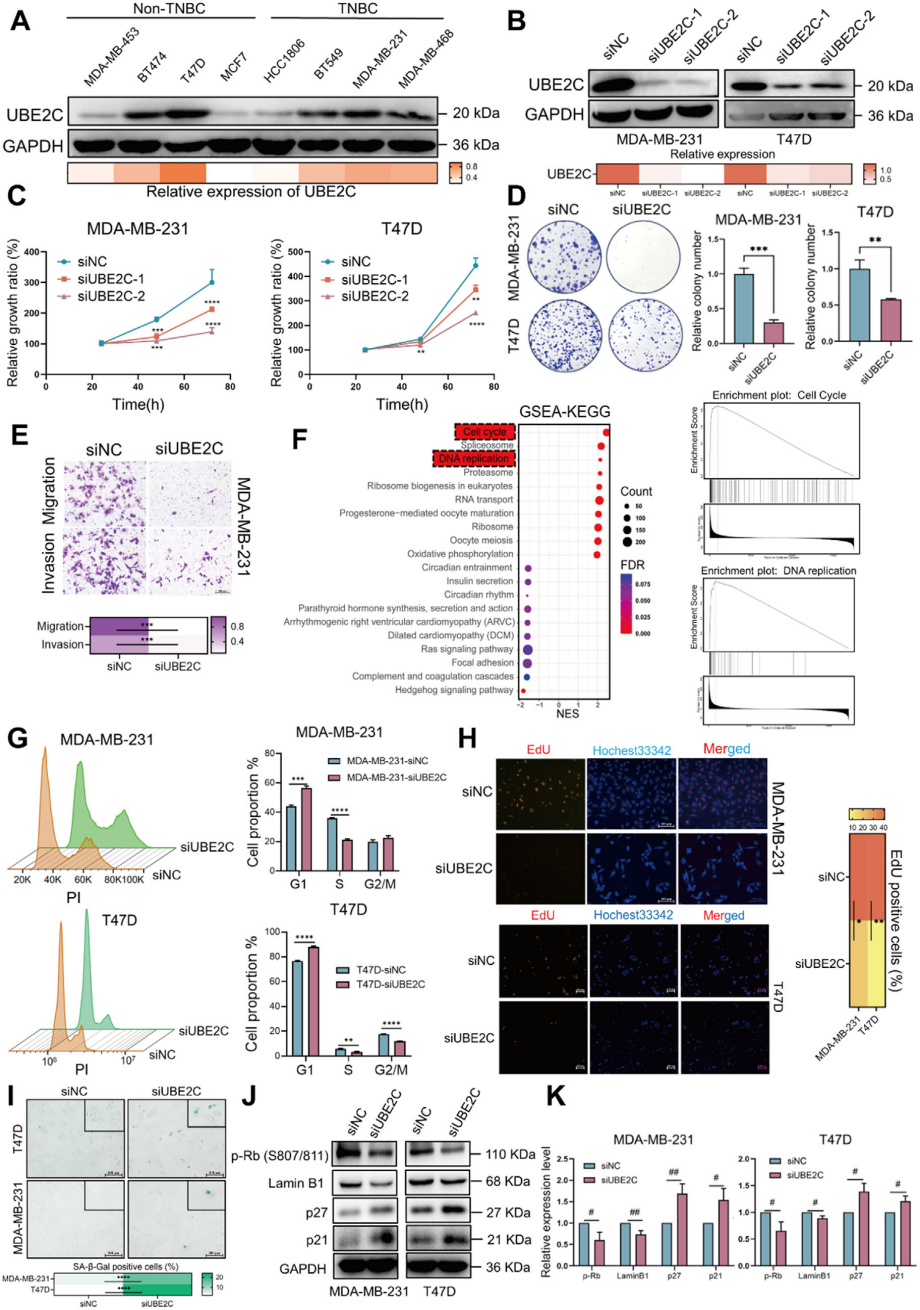
UBE2C knockdown inhibits the proliferation of breast cancer cells and induces cellular senescence. A) The expression of UBE2C in eight breast cancer cell lines was checked by Western blot. (B) After transfection of UBE2C‐specific siRNA for 72 h, the expression of UBE2C was checked by Western blot. (C) After transfection of UBE2C‐specific siRNA for 72 h, CCK‐8 assay was performed to verify the effect of UBE2C knockdown on the proliferation of MDA‐MB‐231 and T47D cells in 24, 48, and 72 h. D) After transfection of UBE2C‐specific siRNA for 72 h, colony formation assay was conducted to verify the effect of UBE2C knockdown on the colony formation ability of MDA‐MB‐231 and T47D cells. E) After transfection of UBE2C‐specific siRNA for 72 h, Transwell assay was performed to verify the effect of UBE2C knockdown on the migration and invasion ability of MDA‐MB‐231 cells. F) GESA enrichment was conducted to analyze the functional enrichment of UBE2C correlated genes in breast cancer against pathways from KEGG using the LinkedOmics database. G) Flow cytometry was used to analyze the cell cycle distribution after UBE2C was knocked down in MDA‐MB‐231 and T47D cells. H) After transfection of UBE2C‐specific siRNA for 72 h, the effect of UBE2C knockdown on the DNA synthesis rate was detected by EdU synthesis assay in MDA‐MB‐231 and T47D cells. I) After transfection of UBE2‐specific siRNA for 72 h, senescent cells were stained with SA‐β‐Gal after UBE2C knockdown in MDA‐MB‐231 and T47D cells. J) After transfection of UBE2C‐specific siRNA for 72 h, Western blot was conducted to validate the expression of senescence‐related protein after UBE2C knockdown in MDA‐MB‐231 and T47D cells. K) The quantification result of Western blot in Figure 2J. Data were replicated for at least 3 times. *P < 0.05, **P < 0.01, ***P < 0.001, ****P < 0.0001 versus siNC group. #*P* < 0.05, ##*P* < 0.01 between two groups.

To explore the biological function of UBE2C in breast cancer, its expression was examined in eight breast cancer cell lines was checked using Western blot. T47D and MDA‐MB‐231 cell lines were chosen for further validation because of their relatively higher UBE2C expression (**Figure** **2**A). Two UBE2C‐specific siRNAs were used to knock down UBE2C in T47D and MDA‐MB‐231 cells (Figure 2B). Knockdown of UBE2C significantly inhibited the proliferation, colony formation, migration, and invasion of breast cancer cells (Figure 2C–E). In contrast, overexpression of UBE2C using the 3×Flag‐UBE2C lentivirus promoted the proliferation and colony formation ability of MCF7 cells (Figure S2A–C, Supporting Information). The LinkedOmics database was used to investigate the molecular signature, biological pathway, and functional role of UBE2C in breast cancer.^[^ ^29^ ^]^ Based on the transcriptomic data from TCGA breast cancer, genes that were positively and negatively correlated with UBE2C were analyzed and plotted in a volcano plot (Figure S3A, Supporting Information). The first 50 genes that were positively correlated with UBE2C and the first 50 genes that were negatively correlated with UBE2C are shown in the heatmap (Figure S3B,C, Supporting Information). Furthermore, GESA was conducted to analyze the functional enrichment of UBE2C‐correlated genes in breast cancer against Gene Ontology (GO) categories and pathways from the Kyoto Encyclopedia of Genes and Genomes (KEGG). GO enrichment showed that the co‐expressed genes were enriched in multiple categories associated with cell cycle and DNA replication (Figure S3D–F, Supporting Information). KEGG analysis also revealed that cell cycle and DNA replication pathways were enriched in UBE2C co‐expressed genes (Figure 2F). Based on the results of GSEA enrichment, we speculated whether the knockdown of UBE2C could affect the cell cycle of breast cancer cells, and flow cytometry was conducted to analyze the cell cycle distribution. As shown in Figure 2G, the cell cycle was significantly arrested at the G0/G1 phase after UBE2C knockdown. In addition, the DNA synthesis rate of T47D and MDA‐MB‐231 cells was significantly reduced after the knockdown of UBE2C expression (Figure 2H). Considering that cell cycle arrest is a typical characteristic of cellular senescence,^[^ ^30^ ^]^ we further validated whether the inhibition of UBE2C could induce senescence in breast cancer. Enhanced senescence associated β‐galactosidase (SA‐β‐Gal) activity is a hallmark of cellular senescence.^[^ ^31^ ^]^ As shown in Figure 2I, the proportion of SA‐β‐Gal positive cells was significantly increased, which also demonstrated that inhibition of UBE2C led to cellular senescence. Moreover, the mRNA level of TNFα and IL1β, the major senescence‐associated secretory phenotype (SASP) factors,^[^ ^32^ ^]^ was significantly upregulated after knockdown of UBE2C expression (Figure S4A,B, Supporting Information). Ablation of UBE2C also resulted in the overexpression of the cell cycle inhibitors p27, p21 and the loss of lamin B1, which further demonstrated the occurrence of senescence (Figure 2J,K).^[^ ^30^ ^]^ In summary, inhibition of UBE2C suppressed proliferation and induced senescence in breast cancer cells.

### 2.3 Inhibition of UBE2C Sensitizes Breast Cancer Cells to Doxorubicin

**Figure 3:**
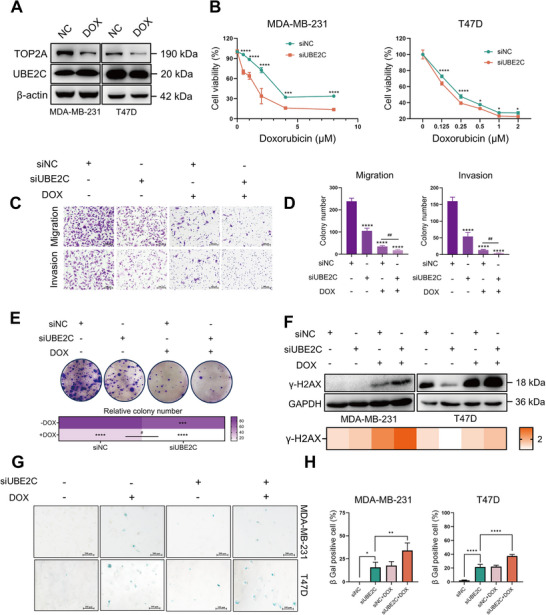
Inhibition of UBE2C sensitizes breast cancer cells to doxorubicin in vitro. A) MDA‐MB‐231 and T47D cells were treated with 0.5 µm doxorubicin for 48 h, and the expression of TOP2A and doxorubicin was verified by Western blot. (B) After transfection of UBE2C‐specific siRNA for 72 h, MDA‐MB‐231 and T47D cells were treated with different concentrations of doxorubicin for 48 h, and cell viability was detected using CCK‐8. C) After transfection of UBE2C specific siRNA for 72 h, MDA‐MB‐231 cells were treated with 0.5 µm doxorubicin for 24 h and Transwell assay was carried out to validate the synergistic effect of UBE2C ablation and doxorubicin treatment on the migration and invasion ability of MDA‐MB‐231 cells. D) The quantification result of Transwell assay in Figure 4C. E) After transfection of UBE2C‐specific siRNA for 72 h, MDA‐MB‐231 cells were treated with 0.5 µm doxorubicin for 24 h and colony formation assay was carried out to validate the synergistic effect of UBE2C ablation and doxorubicin treatment on the colony formation ability of MDA‐MB‐231 cells, and the corresponding quantification result was shown. F) After transfection of UBE2C‐specific siRNA for 72 h, MDA‐MB‐231 cells were treated with 0.5 µm doxorubicin, and Western blot was performed to check the expression of γ‐H2AX. The quantification of the protein level of γ‐H2AX was calculated and shown. G) After transfection of UBE2C‐specific siRNA for 72 h, MDA‐MB‐231 cells were treated with 0.5 µm doxorubicin, SA‐β‐Gal staining was performed to validate the synergistic effect of UBE2C ablation and doxorubicin treatment on inducing senescence in MDA‐MB‐231 and T47D cells. H) The quantification result of SA‐β‐Gal staining in Figure 3G. Data were replicated for at least 3 times. *P < 0.05, **P < 0.01, ***P < 0.001, ****P < 0.0001 versus siNC group. ## *P* < 0.01, #### *P* < 0.0001 between two groups. DOX, doxorubicin.

**Figure 4:**
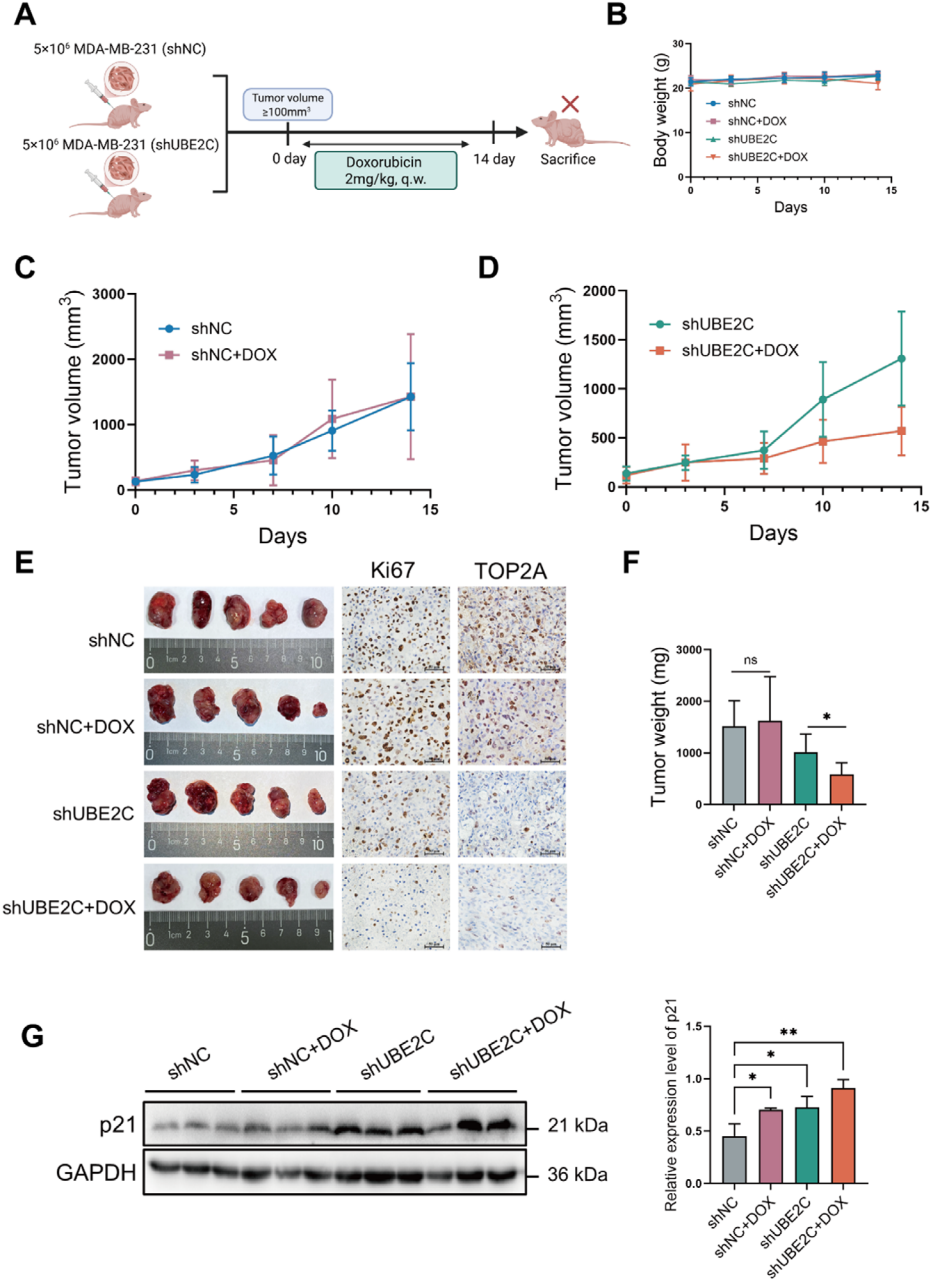
Inhibition of UBE2C sensitizes breast cancer cells to doxorubicin in vivo. A) The scheme of animal experiment. B) Body weight of mice was measured since the first day of administration, and there was no significant change in body weight during doxorubicin administration. C) Tumor volumes of mice in shNC group were recorded twice a week during doxorubicin administration (n = 5). D) Tumor volume of mice in shUBE2C group was recorded twice a week during doxorubicin administration (n = 5). E) After sacrifice, tumor tissues were collected, and the representative image of tumors in shNC and shUBE2C group and the representative IHC results of Ki67 and TOP2A in each group was shown. UBE2C knockdown combined with doxorubicin treatment had the most significant tumor inhibitory effect. F) The average tumor weight in each group was calculated, and the combination of UBE2C knockdown with doxorubicin treatment had the most significant tumor inhibitory effect (n = 5). G) The expression of p21 in tumor tissue was verified by Western blot and the quantification result was shown. The combination of UBE2C knockdown with doxorubicin treatment induced the most significant expression of p21 among each group. *P < 0.05, **P < 0.01 between two groups. DOX, doxorubicin.

Since doxorubicin exerts an anti‐cancer effect by binding to TOP2A and impeding DNA replication, we first verified the effect of doxorubicin on the expression of TOP2A and UBE2C.^[^ ^33^ ^]^ As shown in **Figure** **3**A and Figure S5A (Supporting Information), doxorubicin significantly inhibited the expression of TOP2A but had little effect on the expression of UBE2C in breast cancer cells. Given that UBE2C plays an important role in regulating the cell cycle and senescence in breast cancer cells, we examined whether UBE2C could affect the sensitivity of breast cancer cells to doxorubicin. Interestingly, the cell viability of MDA‐MB‐231 and T47D cells was significantly more reduced after UBE2C knockdown in the doxorubicin treated group (Figure 3B; Figure S5B, Supporting Information). To further confirm the effect of UBE2C knockdown on doxorubicin effects, Transwell and colony formation assays were performed. The Transwell assay showed that both UBE2C knockdown and doxorubicin treatment inhibited the migration and invasion ability of MDA‐MB‐231 cells. Moreover, UBE2C ablation further enhanced the inhibitory effect of doxorubicin on MDA‐MB‐231 cells (Figure 3C,D). The colony formation assay demonstrated that UBE2C knockdown and doxorubicin treatment inhibited the colony formation ability of MDA‐MB‐231 cells and UBE2C knockdown further enhanced the inhibitory effect of doxorubicin on MDA‐MB‐231 cells (Figure 3E). In addition, UBE2C knockdown enhanced the expression of γ‐H2AX induced by doxorubicin (Figure 3F), which is the hallmark of DNA damage.^[^ ^34^ ^]^ Given that doxorubicin can induce senescence,^[^ ^7^ ^]^ we explored the effect of UBE2C knockdown on doxorubicin‐induced senescence. SA‐β‐Gal staining showed that the proportion of doxorubicin‐induced SA‐β‐Gal positive cells was increased after UBE2C knockdown (Figure 3G,H). To further investigate the effect of UBE2C knockdown on doxorubicin sensitivity in vivo, an MDA‐MB‐231 xenograft was established and doxorubicin (2 mg kg^−1^) was administered intraperitoneally (**Figure** **4**A). This treatment had no significant inhibitory effect on body weight (Figure 4B) as well as the on growth of tumors in the shNC group in terms of tumor volume (Figure 4C) and tumor weight (Figure 4E,F). However, UBE2C knockdown sensitized breast cancer tumors to doxorubicin (Figure 4D,F). The IHC results of Ki67 and TOP2A demonstrated that combination of UBE2C knockdown and doxorubicin treatment had the strongest anti‐tumor effect (Figure 4E). Moreover, the expression of senescence marker p21^[^ ^35^ ^]^ was upregulated after UBE2C knockdown and doxorubicin treatment, which also consolidated the effect of UBE2C and doxorubicin on cellular senescence (Figure 4G). Taken together, these results indicated that UBE2C knockdown sensitized breast cancer cells to doxorubicin in vitro and in vivo.

### 2.4 UBE2C Modulates the Stability of TOP2A in a Ubiquitination‐Dependent Manner

**Figure 5:**
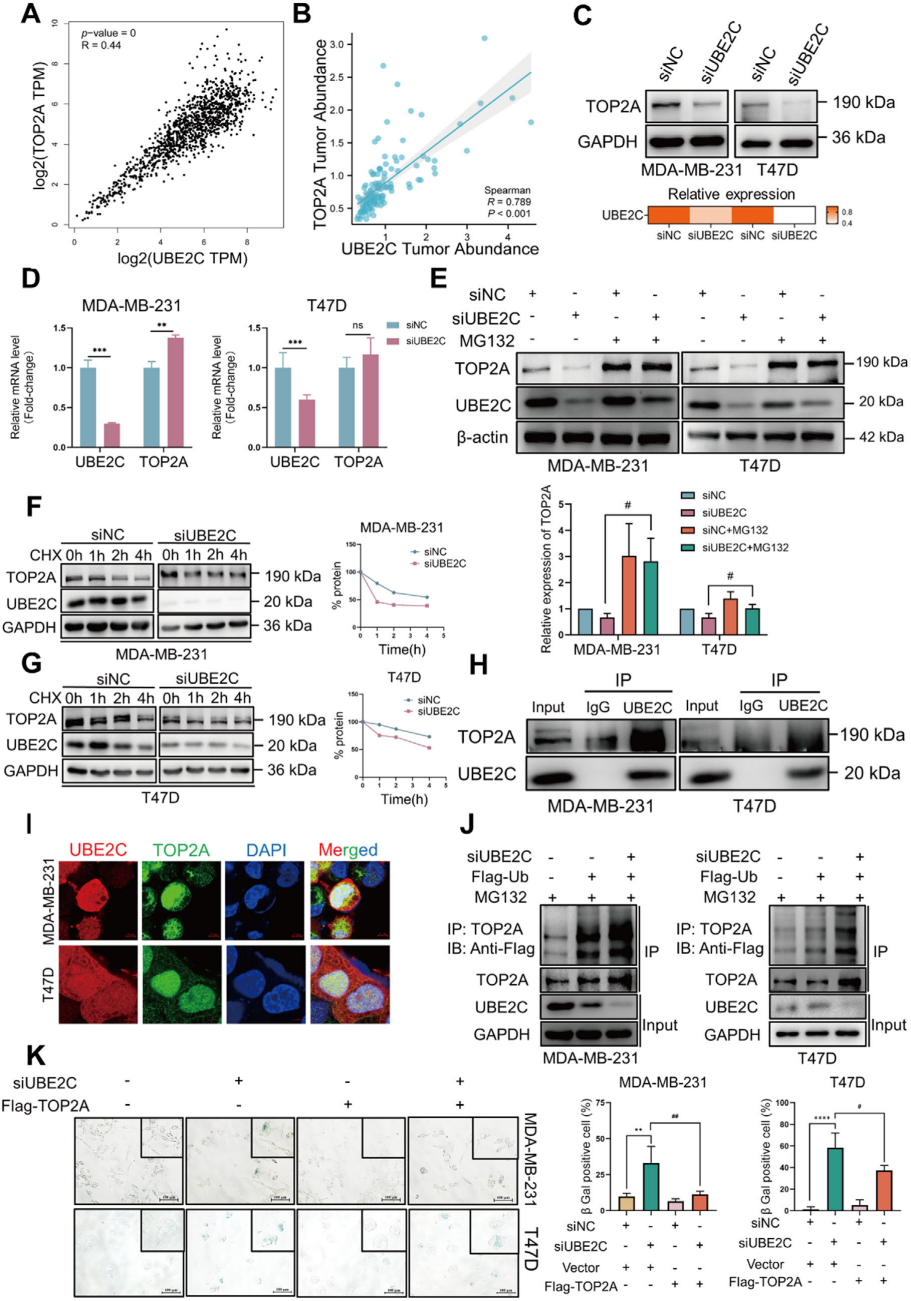
UBE2C modulates the stability of TOP2A via a ubiquitination dependent manner. A) GEPIA database was used to analyze the correlation between the mRNA level of UBE2C and TOP2A in breast cancer using TCGA breast cancer cohorts. B) cProSite database was used to analyze the correlation between the protein level of UBE2C and TOP2A in breast cancer using CPTAC breast cancer cohorts. C) After transfection of UBE2C‐specific siRNA for 72 h, the expression of TOP2A after UBE2C knockdown was checked by Western blot in MDA‐MB‐231 and T47D cells. The quantification result was also displayed. D) After transfection of UBE2C‐specific siRNA for 72 h, the mRNA level of UBE2C and TOP2A after UBE2C knockdown was measured by RT‐qPCR in MDA‐MB‐231 and T47D cells. E) After transfection of UBE2C‐specific siRNA for 72 h, MDA‐MB‐231 and T47D cells were treated with MG132 for 8 h, and the effect of MG132 on the expression of TOP2A was checked by Western blot. The quantification of the protein level of TOP2A was calculated and shown. F) After transfection of UBE2C‐specific siRNA for 72 h, MDA‐MB‐231 cells were treated with 50 µg mL^−1^ CHX for 0, 1, 2, and 4 h, and the effect of UBE2C on the protein stability of TOP2A was checked by Western blot. G) After transfection of UBE2C‐specific siRNA for 72 h, MDA‐MB‐231 cells were treated with 50 µg mL^−1^ CHX for 0, 1, 2, and 4 h, and the effect of UBE2C on the protein stability of TOP2A was checked by Western blot in T47D cells. H) The interaction of UBE2C and TOP2A was checked by co‐immunoprecipitation (Co‐IP) in MDA‐MB‐231 and T47D cells. I) Cell distribution of UBE2C and TOP2A was checked by immunofluorescence in MDA‐MB‐231 cells and T47D cells. J) After transfection of UBE2C specific siRNA for 72 h, Co‐IP was carried out to validate the effect of UBE2C knockdown on the ubiquitination of TOP2A in MDA‐MB‐231 and T47D cells. K) MDA‐MB‐231 and T47D cells were simultaneously transfected with UBE2C‐specific siRNA and Flag‐TOP2A plasmid for 72 h and SA‐β‐Gal staining was performed to verify the effect of overexpression of TOP2A on senescence induced by UBE2C knockdown. Data were replicated for at least 3 times. *P < 0.05, **P < 0.01, ***P < 0.001, ****P < 0.0001. # *P* < 0.05, ## *P* < 0.01 between two groups.

Based on the above results, we investigated whether UBE2C regulated the sensitivity to doxorubicin by affecting the expression of TOP2A, since TOP2A was the direct target of doxorubicin. Bioinformatics analysis was performed to verify the expression level of TOP2A in breast cancer. TOP2A was significantly overexpressed in breast cancer cells at both the mRNA and protein levels (Figure S6A–C, Supporting Information). Moreover, TOP2A expression correlated with poor prognosis in patients with breast cancer patients in relation to OS (Figure S6D, Supporting Information) and RFS (Figure S6E, Supporting Information). The GEPIA and cProSite databases were used to analyze the association between UBE2C and TOP2A, according to mRNA and protein abundance in breast cancer. The mRNA expression of UBE2C positively correlated with that of TOP2A in breast cancer (**Figure** **5**A). Similarly, the protein expression of UBE2C was also positively correlated with that of TOP2A in breast cancer (Figure 5B). Western blot was performed to check the expression of TOP2A after UBE2C knockdown, and the results showed that TOP2A expression was downregulated (Figure 5C). To clarify how UBE2C regulated the expression of TOP2A in breast cancer, the transcriptional level of TOP2A was determined using RT‐qPCR after UBE2C knockdown. However, the mRNA level of TOP2A was not decease after knockdown of UBE2C, demonstrating that UBE2C had no effect on the transcription of TOP2A (Figure 5D). To verify whether UBE2C affected the protein levels of TOP2A, MG132 and BafA1 were used to block the ubiquitin‐proteasome and autophagosome‐lysosome pathways in breast cancer cells. As shown in Figure 5E, UBE2C knockdown downregulated the expression of TOP2A and treatment with MG132 recovered the expression of TOP2A in breast cancer cells. However, treatment with BafA1 had no such effect (Figure S7, Supporting Information). Moreover, UBE2C knockdown reduced the protein stability of TOP2A in breast cancer cells (Figure 5F,G). These results demonstrated that UBE2C might affect the proteasomal degradation of TOP2A. UBE2C also endogenously interacted with TOP2A (Figure 5H) and immunofluorescence analysis showed that UBE2C colocalized with TOP2A, especially in the nucleus (Figure 5I). Moreover, UBE2C knockdown promoted the ubiquitination of TOP2A in breast cancer cells (Figure 5J). Overexpression of TOP2A could not only reverse cell growth inhibition caused by UBE2C knockdown but also by doxorubicin treatment (Figure S6F,G, Supporting Information), and also alleviated cell senescence induced by UBE2C knockdown, which was reflected by SA‐β‐Gal staining (Figure 5K). Taken together, these results indicated that UBE2C modulated TOP2A expression via a ubiquitination‐dependent manner.

### 2.5 UBE2C Knockdown Promotes Parkin‐Mediated K63‐Linked Ubiquitination of TOP2A

**Figure 6:**
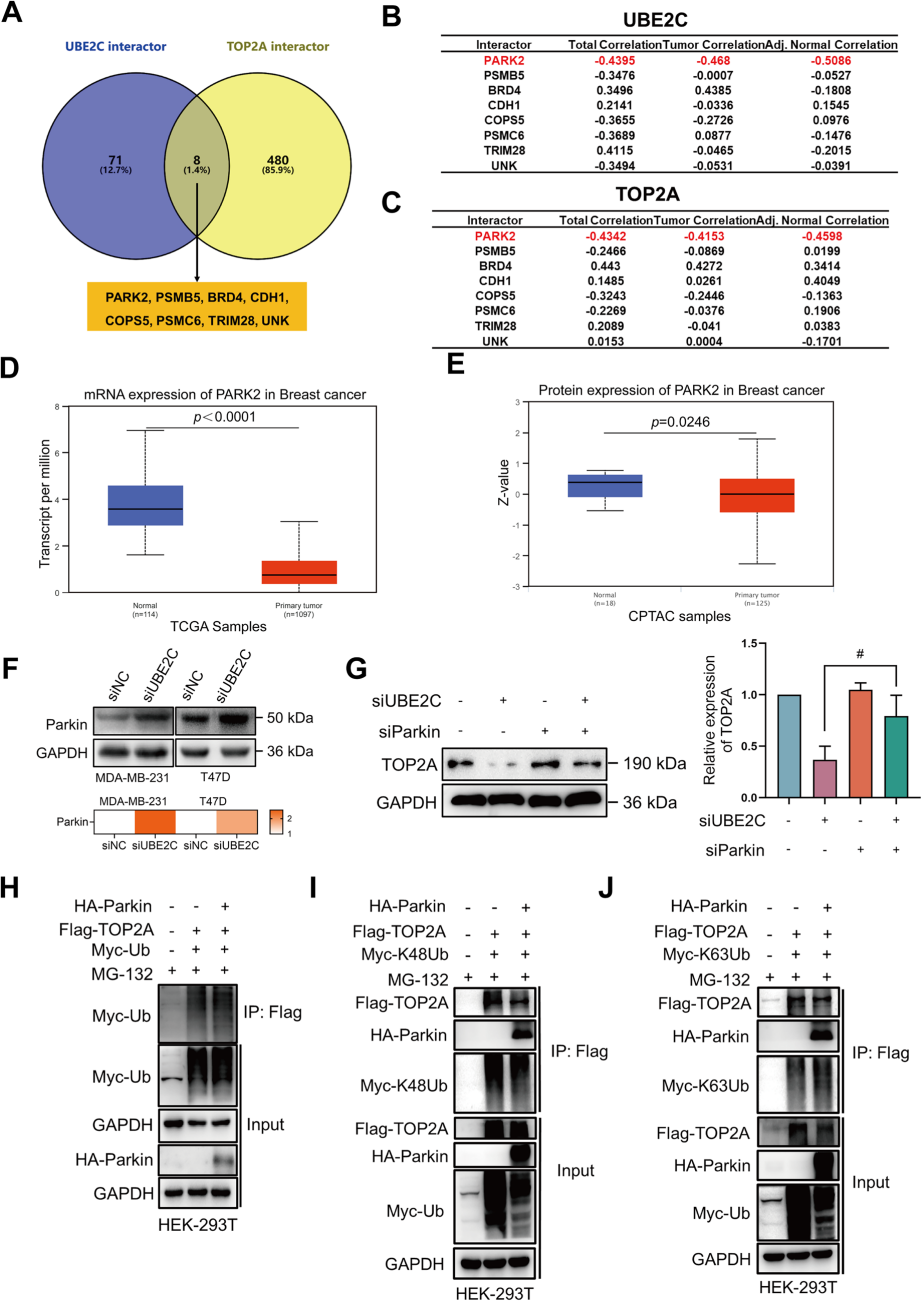
UBE2C knockdown promotes Parkin‐mediated K63‐linked ubiquitination of TOP2A. A) BioGRID database was used to analyze the interactors of UBE2C and TOP2A, and the common interactors was filtered. B) cProSite database was used to analyze the correlation of the protein level of UBE2C and eight common interactors in Figure 6A using CPTAC breast cancer cohort. C) cProSite database was used to analyze the correlation of the protein level of UBE2C and eight common interactors in Figure 6A using CPTAC breast cancer cohort. D) The mRNA level of PARK2 in breast cancer (n = 1097) and normal tissues (n = 114) was analyzed by UALCAN database. E) The protein level of PARK2 in breast cancer (n = 125) and normal tissues (n = 18) was analyzed by UALCAN database. F) After transfection of UBE2C specific‐siRNA for 72 h, the effect of UBE2C knockdown on the expression of parkin in MDA‐B‐231 and T47D cells was checked by Western blot. The quantification of the protein level of PARK2 was calculated and shown. G) After transfection of UBE2C and Parkin‐specific siRNA for 72 h, the effect of Parkin knockdown on the expression of TOP2A after UBE2C knockdown was verified by Western blot. The quantification of the protein level of TOP2A was calculated and shown. H) HEK293T cells were transfected with HA‐Parkin, Flag‐TOP2A, and Myc‐Ub plasmids for 24 h, and the effect of Parkin on the ubiquitination of TOP2A was verified by Co‐IP. I) HEK293T cells were transfected with HA‐Parkin, Flag‐TOP2A, and Myc‐K48Ub plasmids for 24 h, and the effect of Parkin on the K‐48 linked ubiquitination of TOP2A was verified by Co‐IP. J) HEK293T cells were transfected with HA‐Parkin, Flag‐TOP2A, and Myc‐K63Ub plasmids for 24 h, the effect of parkin on the K63‐linked ubiquitination of TOP2A was verified by Co‐IP. Data were replicated for at least 3 times. # *P* < 0.05 between two groups.

To further clarify the regulatory mechanism of UBE2C on the ubiquitination of TOP2A, the E3 ubiquitin ligases responsible for TOP2A ubiquitination were sorted and validated. The BioGRID database was used to analyze the possible common interactors between UBE2C and TOP2A. There were 71 reported interactors of UBE2C and 480 reported interactors of TOP2A (**Figure** **6**A). Eight common interactors between UBE2C and TOP2A were identified, including PARK2, proteasome 20S subunit beta 5 (PSMB5), bromodomain‐containing protein 4 (BRD4), E ‐cadherin (CDH1), the fifth component of the constitutive photomorphogenic‐9 signalosome (COPS5), proteasome 26S aubunit, ATPase 6 (PSMC6), the E3 SUMO ligase tripartite motif‐containing protein 28 (TRIM28), and UNK. Most of them belong to the E3 ubiquitin ligase family. To further analyze the association of these common interactors with UBE2C and TOP2A, cProSite database was used to analyze the protein correlation between UBE2C, TOP2A, and the eight common interactors (Figure 6B,C). Among them, PARK2, which encodes Parkin, ranked first as the most negatively correlated interactor between UBE2C and TOP2A at the protein level. In breast cancer, Parkin was significantly downregulated both at the mRNA and protein level (Figure 6D,E). Moreover, UBE2C knockdown upregulated the expression of Parkin (Figure 6F). To verify how Parkin affected the expression of TOP2A, UBE2C and Parkin were simultaneously knocked down, and the expression of TOP2A was assessed using Western blot. As shown in Figure 6G, Parkin knockdown reversed the downregulation of TOP2A caused by UBE2C knockdown. To further demonstrate the effect of Parkin on the ubiquitination of TOP2A, co‐immunoprecipitation (Co‐IP) was performed to validate the ubiquitin level of TOP2A. Parkin not only enhanced the overall ubiquitin level of TOP2A but also promoted the K63‐linked ubiquitin of TOP2A but not the K48‐linked ubiquitin of TOP2A (Figure 6H–J). Taken Altogether, these results showed that UBE2C knockdown promoted Parkin‐mediated K63‐linked ubiquitination of TOP2A.

### 2.6 FOXM1 Transcriptionally Regulates the Expression of UBE2C in Breast Cancer

**Figure 7:**
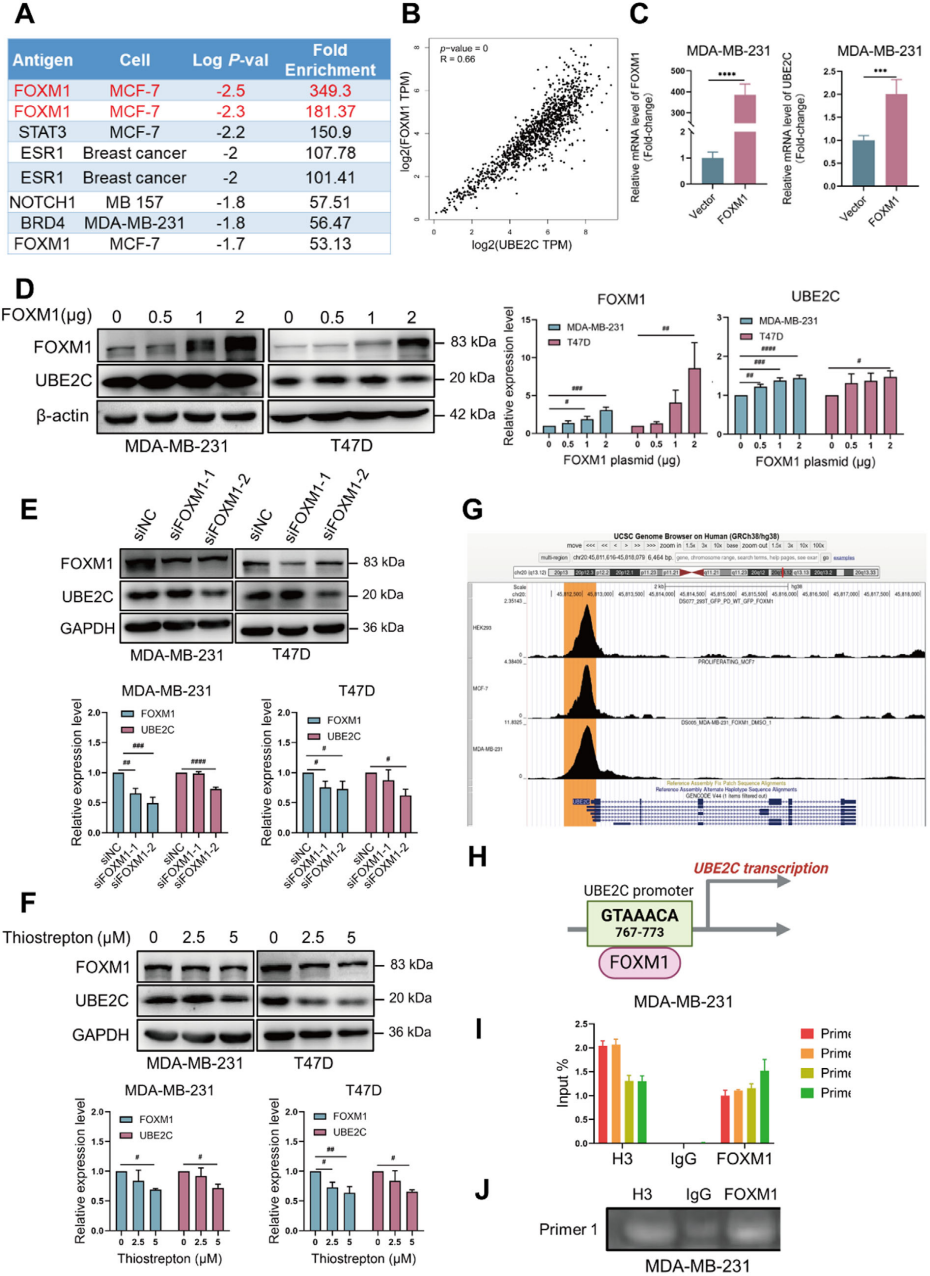
FOXM1 transcriptionally regulates the expression of UBE2C in breast cancer. A) ChIP‐Atlas database was used to analyze the potential transcription factor of FOXM1 in breast cancer. B) GEPIA database was used to analyze the correlation of mRNA level of UBE2C and FOXM1 based on the data from the TCGA breast cancer cohort. C) After transfection of FOXM1 plasmid for 24 h, RT‐qPCR was used to analyze the mRNA level of FOXM1 and UBE2C after overexpression of FOXM1 in MDA‐MB‐231 cells. D) After transfection of 0, 0.25, 1, and 2 µg of FOXM1 plasmid for 24 h, the expression of FOXM1 and UBE2C was checked by Western blot. The quantification of the protein level of FOXM1 and UBE2C was calculated and shown. E) The expression of FOXM1 and UBE2C was checked by Western blot after FOXM1 was knocked down after transfection of FOXM1‐specific siRNAs for 72 h in MDA‐MB‐231 cells and T47D cells. The quantification of the protein level of FOXM1 and UBE2C were calculated and shown. F) MDA‐MB‐231 cells and T47D cells were treated with 0, 2.5, and 5 µm thiostrepton for 24 h and the expression of FOXM1 and UBE2C was checked by Western blot. The quantification of the protein level of FOXM1 and UBE2C was calculated and shown. G) UCSC Genome Browser was used to analyze the potential binding site of FOXM1 on the promoter of UBE2C. H) JASPAR database was used to analyze the FOXM1 binding site location and sequence in the UBE2C promoter. I) ChIP‐qPCR was performed to validate the binding of FOXM1 on the promoter of UBE2C. J) Gel electrophoresis of the product of ChIP‐qPCR. Data were replicated for at least 3 times. ***P < 0.001, ****P < 0.0001 versus Vector group. # *P* < 0.05, ## *P* < 0.01, ### *P* < 0.001, #### *P* < 0.0001 between two groups.

To further explore the transcriptional regulatory mechanisms of UBE2C in breast cancer, the ChIP‐Atlas database was used to identify potential transcriptional factors of UBE2C in breast cancer. As shown in **Figure** **7**A, FOXM1 was identified as the most likely transcriptional factor of UBE2C in breast cancer cells. FOXM1 expression also positively correlated with UBE2C expression at the transcriptional level (Figure 7B). Overexpression of FOXM1 could upregulate UBE2C expression at the mRNA level and protein levels in breast cancer cells (Figure 7C,D). Conversely, knockdown of FOXM1 downregulated the expression of UBE2C in breast cancer cells (Figure 7E). Moreover, thiostrepton, a FOXM1 selective inhibitor, inhibited the expression of FOXM1 and UBE2C in a dose‐dependent manner (Figure 7F). Using USCS Genome Browser to analyze FOXM1 chromatin immunoprecipitation (ChIP)‐seq data on the UBE2C promoter, a strong FOXM1 binding peak of could be observed in the promoter region of UBE2C (Figure 7G). The potential binding site location and sequence of FOXM1 in UBE2C promoter are depicted in Figure 7H. Finally, ChIP‐PCR was performed to confirm that FOXM1 directly bound to the promoter region of UBE2C (Figure 7I,J). Taken together, these results indicated that FOXM1 transcriptionally regulated the expression of UBE2C in breast cancer cells.

## 3 Discussion

Anthracycline plus taxane‐based chemotherapy is a major therapeutic approach for breast cancer.^[^ ^4^ ^]^ As one of the most commonly used anthracyclines, doxorubicin is the core component of breast cancer chemotherapy regimens.^[^ ^36^ ^]^ However, therapeutic resistance to doxorubicin restricts its clinical efficacy and causes chemotherapy failure.^[^ ^37^ ^]^ Therefore, there is an urgent need to identify novel therapeutic targets to overcome doxorubicin resistance.

**Figure 8:**
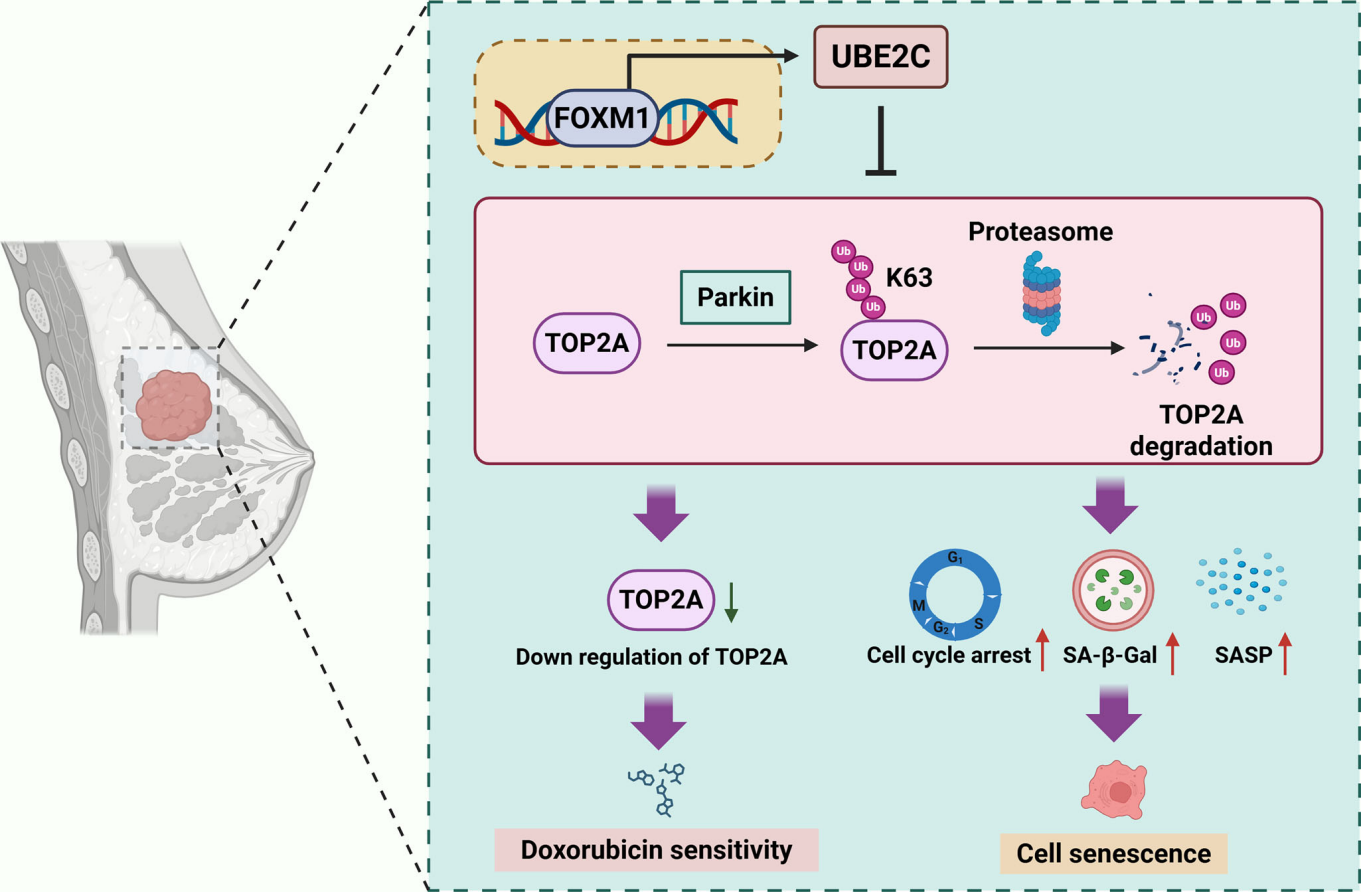
The mechanism of the tumorigenesis role of UBE2C in breast cancer. In breast cancer, UBE2C is significantly upregulated and transcriptionally regulated by FOXM1. UBE2C regulates the protein level of TOP2A by affecting Parkin‐mediated K63‐linked ubiquitination of TOP2A, thus modulating cellular senescence and sensitivity of doxorubicin in breast cancer. (The figure was created by BioRender.).

Although the oncogenic role of UBE2C in breast cancer has been partially elucidated, the exact mechanisms and downstream signaling of UBE2C remain unclear. In the present study, we found that UBE2C was aberrantly upregulated in breast cancer and correlated with poor prognosis in patients with breast cancer. UBE2C inhibition suppressed breast cancer cell proliferation. Interestingly, the inhibition of UBE2C could also induce senescence in breast cancer cells, which was first reported by our group. Moreover, UBE2C inhibition sensitized breast cancer cells to doxorubicin by downregulating the expression of TOP2A. Mechanistically, UBE2C inhibition promoted Parkin‐mediated K63‐linked ubiquitination of TOP2A and led to its proteasomal degradation. The expression of UBE2C was transcriptionally regulated by FOXM1 in breast cancer cells. Taken together, these results showed that inhibition of UBE2C could promote Parkin‐mediated K63‐linked ubiquitination of TOP2A to induce senescence and sensitize breast cancer cells to doxorubicin (**Figure** **8**).

As the direct target of doxorubicin, the expression level of TOP2A determines the efficacy of doxorubicin in chemotherapy.^[^ ^38^ ^]^ The expression of TOP2A is controlled at both the transcriptional and post‐translational levels. At the transcriptional level, it has been reported that the translation of TOP2A could be regulated by the RNA‐binding protein HuR and E2F transcription factor 1 (E2F1).^[^ ^38^, ^39^ ^]^ Post‐translational modifications also influence the protein abundance of TOP2A. In doxorubicin‐resistant breast cancer cells, OGT‐catalyzed hyper‐O‐GlcNAcylation of TOP2A enhanced its catalytic activity and promoted the interaction between TOP2A and cell cycle regulators, thus contributing to progression of breast cancer progression and doxorubicin resistance.^[^ ^40^ ^]^ Moreover, ubiquitin modification of TOP2A is not only involved in regulating the protein stability of TOP2A but also affects drug sensitivity to TOP2A inhibitors.^[^ ^41^, ^42^ ^]^ It is acknowledged that doxorubicin stabilizes the TOP2A complex by binding to the double‐strand DNA breaks and the expression level of TOP2A determines the efficacy of doxorubicin.^[^ ^43^ ^]^ As TOP2A is overexpressed in breast cancer, treatment with doxorubicin significantly inhibited the expression of TOP2A in MDA‐MB‐231 and T47D cells (Figure 3A; Figure S5A, Supporting Information). Moreover, overexpression of TOP2A reversed the inhibitory effect of doxorubicin on cell growth (Figure S6G, Supporting Information). The above results demonstrated that doxorubicin exerted its anti‐tumor effect by downregulating TOP2A. Since we found that knockdown of UBE2C reduced the expression of TOP2A, we speculated that inhibiting UBE2C might have a synergistic effect with doxorubicin, enhancing its ability to inhibit TOP2A expression and reach a better anti‐tumor effect. In the present study, UBE2C was identified for the first time as a novel regulator of TOP2A expression in breast cancer. The protein abundance of UBE2C positively correlated with that of TOP2A and UBE2C knockdown inhibited the expression of TOP2A by promoting its ubiquitination and proteasomal degradation. Moreover, UBE2C inhibition also sensitized breast cancer cells to doxorubicin by downregulating TOP2A and aggravating doxorubicin‐induced cellular senescence. Thus, we hypothesized that UBE2C knockdown sensitized breast cancer cells to doxorubicin through the synergistic inhibition of TOP2A. These data emphasized that UBE2C could serve as a potential target to overcome doxorubicin resistance in breast cancer.

As an E3 ubiquitin ligase, Parkin is well known as the co‐mediator with PTEN‐induced kinase‐1 (PINK1) in regulating mitophagy.^[^ ^44^ ^]^ Parkin also plays functional roles in multiple cellular processes including cell proliferation, cell cycle, apoptosis, necroptosis, and metastasis.^[^ ^45^ ^]^ In cancer, Parkin functions as a tumor suppressor, but the exact mechanism remained unclear until researchers discovered that Parkin prevented cancer progression by inhibiting necrosis.^[^ ^46^ ^]^ In breast cancer, Parkin directly interacts with BRCA1 and HIF1α, mediating their degradation to function as a tumor suppressor.^[^ ^47^, ^48^ ^]^ Parkin also enhances breast cancer sensitivity to paclitaxel by increasing the interaction between paclitaxel and microtubules.^[^ ^49^ ^]^ Although UBE2C could regulate the protein level of TOP2A through ubiquitination, UBE2C was not directly responsible for the degradation of TOP2A because both had the same changes at the protein level. To identify the mediator of TOP2A ubiquitination, the BIOGRID database was used to analyze the common interactors of UBE2C and TOP2A. Among these interactors, Parkin ranked first as an E3 ubiquitin ligase with the most significant negative correlation with the protein levels of UBE2C and TOP2A, indicating that Parkin could mediate the ubiquitination of TOP2A. In the present study, Parkin was first identified to be upregulated after UBE2C was knocked down and functioned as the E3 ubiquitin ligase responsible for the K63‐linked ubiquitination of TOP2A, which not only emphasized the tumor‐suppressive role of Parkin in breast cancer but also clarified the mechanism by which UBE2C affected breast cancer cell sensitivity to doxorubicin.

As a pivotal E2 ubiquitin‐conjugating enzyme, UBE2C interacts with E3 ubiquitin ligase APC/C to catalyze K11‐linked ubiquitination of securin and cyclin B1 to promote their proteasomal degradation, thus facilitating the mitotic transition from metaphase to anaphase.^[^ ^50^, ^51^, ^52^ ^]^ Apart from securin and cyclin B1, there are >50 reported substrate proteins of APC/C and most of them are involved in regulating the cell cycle.^[^ ^20^ ^]^ A recent study revealed that TOP2A was a candidate APC substrate during neuronal differentiation but whether APC/C catalyzed K11‐linked ubiquitination of TOP2A was not clarified.^[^ ^53^ ^]^ However, APC/C did not seem to be responsible for the ubiquitination of TOP2A after UBE2C knockdown in our study, since knockdown of UBE2C led to the dysfunction of APC/C as UBE2C was responsible for the initiation of K11 ubiquitin chains. It was previously reported that Parkin‐mediated K48‐linked ubiquitination promotes the proteasomal degradation of substrate proteins, while Parkin‐mediated K63‐linked ubiquitination leads to protein degradation via the autophagy‐lysosome system and promotes mitophagy.^[^ ^54^, ^55^, ^56^ ^]^ In our study, inhibition of UBE2C upregulated the expression of Parkin to promote K63‐linked ubiquitination of TOP2A. However, elevated K63‐linked ubiquitin did not promote autophagy‐lysosome degradation but rather the proteasomal degradation of TOP2A. The exact mechanisms behind this phenomenon remain unclear and further exploration is needed in the future.

## 4 Conclusion

In the present study, UBE2C was significantly upregulated in breast cancer and was transcriptionally regulated by FOXM1. UBE2C inhibition suppressed the proliferation and induced senescence in breast cancer cells. Moreover, the inhibition of UBE2C downregulated the expression of TOP2A to sensitize breast cancer cells to doxorubicin and aggravate doxorubicin‐induced senescence. Mechanistically, UBE2C inhibition promoted Parkin‐mediated K63‐linked ubiquitination of TOP2A and led to its proteasomal degradation. In vivo, inhibition of UBE2C sensitized breast cancer cells to doxorubicin. Our study revealed that UBE2C is a critical regulator in breast cancer cell proliferation, senescence, and sensitivity to doxorubicin.

## 5 Experimental Section

### Bioinformatic Analysis

The Oncomine (www.oncomine.org/)^[^ ^57^ ^]^ and Tumor Immune Estimation Resource (TIMER) (http://timer.cistrome.org/)^[^ ^58^ ^]^ databases were used to analyze the expression of UBE2C in various cancers. Gene Expression Profiling Interactive Analysis (GEPIA) (http://gepia.cancer‐pku.cn/)^[^ ^59^ ^]^ and UALCAN (ualcan.path.uab.edu/)^[^ ^60^ ^]^ databases were used to analyze UBE2C and TOP2A expression in breast cancer. The Kaplan–Meier plotter (https://www.kmplot.com/analysis/)^[^ ^61^ ^]^ database was used to analyze the correlation between UBE2C and TOP2A expression and prognosis of patients with breast cancer. The Human Protein Atlas (HPA) (https://www.proteinatlas.org/)^[^ ^62^ ^]^ database was used to obtain immunohistochemically stained tissue images of UBE2C in breast cancer and normal breast tissues. The LinkedOmics (http://www.linkedomics.org)^[^ ^29^ ^]^ database was used to perform GSEA. The BioGRID (https://thebiogrid.org) database was used to analyze the interactors of UBE2C and TOP2A.^[^ ^63^ ^]^ The ChIP‐Atlas (https://chip‐atlas.org/) database was used to identify potential transcription factors of UBE2C in breast cancer.^[^ ^64^ ^]^ Finally, JASPAR (https://jaspar.elixir.no/) database was used to analyze FOXM1 binding site location and sequence in the UBE2C promoter.^[^ ^65^ ^]^

### Reagents and Antibodies

Detailed information on the reagents and antibodies used in this study is provided in Tables S1 and S2 (Supporting Information).

### Cell Culture

Human breast cancer cell lines MDA‐MB‐231, MDA‐MB‐468, MDA‐MB‐453, and MCF7 were cultured in DMEM (PM150210, Procell, Wuhan, China) with 10% fetal bovine serum (FBS, 164210–50, Procell, Wuhan, China) and 1% penicillin‐streptomycin (P1400, Solarbio, Beijing, China). Human breast cancer cell lines BT474, T47D, HCC1806, and BT549 were cultured in RPMI‐1640 (PM150110, Procell, Wuhan, China) with 10% FBS and 1% penicillin‐streptomycin (P1400, Solarbio, Beijing, China). All cell lines were purchased from the American Type Culture Collection (ATCC) and cultured in a humidified incubator at 5% CO_2_.

### Cell Viability Assay

Cells were seeded in a 96‐well plate at a density of 3000 per well. After the designed treatment, 10 µL of CCK‐8 regent (No. C0005, TargetMol, Shanghai, China) was added into each well and absorption at 450 nm was measured.

### Transwell Assay

For migration assays, cells were suspended in FBS‐free DMEM and 400 µL of the cell suspension (1×10^5^ cells/mL) was seeded into the upper chamber of Transwell Permeable Supports (3342, Corning Costar, Kennebunk, USA). The lower chamber was filled with DMEM containing 20% FBS. After incubation for 24 h, cells that migrated through the polycarbonate membrane were fixed and stained with 1% crystal violet (G1062, Solarbio, Beijing, China). For invasion assay, Matrigel (354 234, Corning Costar, Kennebunk, USA) was diluted at a 1:7 ratio and coated onto the upper surface of the membrane. The remaining steps were the same as those used for the migration assay.

### Colony Formation

Cells were seeded into 6‐well plates at a density of 1000 cells per well. After culture for 2 weeks, cell colonies were fixed with 4% paraformaldehyde and then stained with 1% crystal violet. Cell colonies were captured, and representative images were shown.

### Cell Cycle Analysis

Cells were fixed in 75% ethanol at 4 °C overnight and washed with PBS three times. Cells were then stained with propidium iodide (PI) solution in the dark for 10 min. Cell cycle was analyzed through flow cytometry using BD FACSVerse Cytometer (BD, San Diego, CA, USA) and Flow‐Jo‐V10.

### EdU DNA Synthesis Assay

BeyoClick EdU Cell Proliferation Kit with Alexa Fluor 555 (C0075S, Beyotime, Shanghai, China) was used to detect the DNA synthesis rate of breast cancer cells according to the manufacturer's instructions. Briefly, cells were incubated with 10 µm EdU for 4 h and then fixed and permeabilized. Cells were then stained with a click reaction solution, and the nuclei were stained with Hoechst 33342. Representative images of EdU‐positive cells were visualized using a fluorescence microscope.

### SA‐β‐Gal Staining Assay

Senescent cells were stained using the Senescence β‐Galactosidase Staining Kit (C0602, Beyotime, Shanghai, China) according to the manufacturer's instructions. In brief, cells were fixed with 4% paraformaldehyde and then stained with SA‐β‐Gal staining working solution overnight at 37 °C. Representative images of SA‐β‐Gal positive cells were captured and visualized.

### Western Blot

Cells were collected and lysed using RIPA lysis buffer (C1053‐100, Gene‐Protein Link, Beijing, China) at 4 °C for 30 min and then centrifuged at 12 000 rpm for 15 min. The supernatant was collected, and protein concentration was quantified using the BCA Protein Assay Kit (P0010, Beyotime, Shanghai, China). The supernatant was denatured with SDS‐PAGE loading buffer (P0015, Beyotime, Shanghai, China) and equal amounts of protein (20 µg per sample) were separated by SDS‐PAGE. Then, the proteins were transferred to polyvinylidene fluoride (PVDF) membranes (IPVH00010, Millipore, Darmstadt, Germany) and blocked with 5% skim milk. After incubation with primary antibodies and secondary antibodies, the bands intensities on the membranes were visualized using Super ECL Detection Reagent (36208ES60, YEASEN, Shanghai, China).

### RNA Extraction and RT‐qPCR

The total RNA of cells was extracted using the RNAeasy Animal RNA Isolation Kit with Spin Column (R0027, Beyotime, Shanghai, China) and SuperRT cDNA Synthesis Kit (CW0741M, CWBIO, Beijing, China) was used for complementary DNA (cDNA) synthesis. qPCR analysis was performed using MagicSYBR Mixture (CW3008M, CWBIO, Beijing, China) according to the manufacturer's instructions. The sequences of primers are listed in Table S3 (Supporting Information). The primers were synthesized by Tsingke Biotechnology Co. Ltd.

### Immunofluorescence

After being fixed and permeabilized, cells were blocked with 5% bovine serum albumin (BSA, A8020, Solarbio, Beijing, China). The cells were then incubated with primary antibodies at 4 °C overnight and incubated with secondary antibodies at room temperature. The nucleus was stained with DAPI (C0065, Solarbio, Beijing, China). Cells were then observed under a laser confocal microscope (Leica TCS SP8X, Leica, Germany).

### Chromatin Immunoprecipitation (ChIP)‐PCR

Cells were collected and processed using the SimpleChIP Enzymatic Chromatin IP Kit (Magnetic Beads) (9003, Cell Signaling Technology, Danvers, MA, USA). Proteins were crosslinked to DNA using 1% formaldehyde. Nuclei were pelleted by centrifugation and digested using micrococcal nuclease. The digested chromatin was incubated overnight at 4 °C with rotation using antibodies against FOXM1, Histone H3, or IgG. ChIP‐grade Protein G magnetic beads were then added to each IP reaction and incubated for 2 h at 4 °C with rotation. Finally, the chromatin precipitate was eluted from the magnetic beads, purified using spin columns, and used as the template for RT‐qPCR analysis.

### Animal Experiments

All animal experiments were conducted in accordance with the National Research Council's Guide for the Care and Use of Laboratory Animals and approved by the Institute of Materia Medica, Chinese Academy of Medical Science and Peking Union Medical College (Beijing, China). To construct a xenograft model in nude mice, 5×10^6^ MDA‐MB‐231‐shNC and MDA‐MB‐231‐shUBE2C cells were harvested and subcutaneously implanted into the right flank of nude mice (female, 17–19 g, Charles River, Beijing, China). When the tumor volume reached 100 mm^3^, the mice were randomly divided into four groups (n = 5): shNC, shUBE2C, shNC with doxorubicin, and shUBE2C with doxorubicin groups. The mice were intraperitoneally administered PBS or doxorubicin (2 mg kg^−1^) once a week. The tumor volume (V, mm^3^) was calculated according to V = 0.5 × l × w^2^ (l = length, w = width in mm). After sacrifice, the tumor tissues were collected, weighed, and photographed.

### Immunohistochemistry (IHC)

Paraffin‐embedded sections of the tumor tissues were dried, deparaffinized, and then immersed in boiling 10 mm sodium citrate buffer (pH 6.0) for 20 min for antigen retrieval. Next, sections were incubated with 3% hydrogen peroxide for 10 min to block endogenous peroxidase activity and blocked with 5% normal goat serum for 20 min. Then, sections were incubated with a primary antibody against Ki67 (GB111141‐100, Servicebio, 1:3000 diluted) and TOP2A (sc‐365916, Santa Cruz, 1:50 diluted) overnight at 4 °C. The next day, sections were incubated with biotinylated secondary antibodies for 1 h and stained with diaminobenzidine (DAB). Cells were counterstained with hematoxylin, and representative images of the stained sections were captured using a microscope (Nikon, Tokyo, Japan).

### Statistical Analysis

Data are presented as mean ± standard deviation (SD). Unpaired Student's t‐test or one‐way analysis of variance (ANOVA) was used to calculate the statistical significance between two or multiple groups. Statistical analyses were performed using GraphPad Prism 10 (GraphPad Software Inc., San Diego, CA, USA), and statistical significance was set at *P* < 0.05.

## Conflict of Interest

The authors declare no conflict of interest.

## Supporting information



Supporting Information

## Data Availability

The data that support the findings of this study are available from the corresponding author upon reasonable request.
