# Supplementary material for: Inhibition of UBE2C Promotes Parkin‐Mediated K63‐Linked Ubiquitination of TOP2A to Induce Senescence and Increase Sensitivity of Doxorubicin in Breast Cancer
Source: Adv Sci (Weinh). 2025 Jul 29;12(32):e17348. doi: 10.1002/advs.202417348 (PMC12407369; doi:10.1002/advs.202417348)
Supplement: Supplementary file 1 — Supporting Information [file ADVS-12-e17348-s001.docx]

Supporting information

**Inhibition of UBE2C promotes Parkin-mediated K63-linked ubiquitination of TOP2A to induce senescence and increase sensitivity of doxorubicin in breast cancer**

*Yihui Yang, Wan Li, Hong Yang, Fang Xu, Sen Zhang, Wanxin Cao, Xiaoxue Li, Xu Zhang, Xiangyin Chi, Hongquan Wang, Guanhua Du, Yumin Wang,* and Jinhua Wang**

Y. Yang, W. Li, H. Yang, F. Xu, S. Zhang, W. Cao, X. Li, X. Zhang, X. Chi, G. Du, J. Wang

The State Key Laboratory of Bioactive Substance and Function of Natural Medicines

Beijing 100050, China

E-mail: wjh@imm.ac.cn

Y. Yang, W. Li, H. Yang, F. Xu, S. Zhang, W. Cao, X. Li, X. Zhang, X. Chi, G. Du, J. Wang

Beijing Key Laboratory of Innovative Drug Discovery and Polymorphic Druggability Research for Cerebrovascular Diseases

Institute of Materia Medica

Chinese Academy of Medical Science and Peking Union Medical College

Beijing 100050, China

H. Wang

Department of Geriatrics

Aerospace Center Hospital

Peking University Aerospace School of Clinical Medicine

Beijing 100049, China.

Y. Wang

Department of Respiratory and Critical Care Medicine

Aerospace Center Hospital

Peking University Aerospace School of Clinical Medicine

Beijing 100049, China.

E-mail: 721wangym@aliyun.com

**Supplementary Table S1. The detailed information of reagents used in the study.**

| **Reagents** | **Company** | **Catalogue** | **City and Country** |
| --- | --- | --- | --- |
| Doxorubicin | MedChemExpress | HY-15142A | Shanghai, China |
| MG-132 | MedChemExpress | HY-13259 | Shanghai, China |
| Cycloheximide | MedChemExpress | HY-12320 | Shanghai, China |
| Thiostrepton | MedChemExpress | HY-B0990 | Shanghai, China |

**Supplementary Table S2. The detailed information of antibodies used in the study.**

| **Antibody** | **Dilution rate** | **Catalogue** | **Company** |
| --- | --- | --- | --- |
| UBE2C | 1:1500 | 66087-1-Ig | Proteintech |
| CyclinB1 | 1:1000 | 55004-1-AP | Proteintech |
| CDK2 | 1:1000 | 18048S | CST |
| CDK4 | 1:1000 | 12790S | CST |
| CDK6 | 1:1000 | 3136S | CST |
| GAPDH | 1:2000 | 60004-1-Ig | Proteintech |
| β-actin | 1:1500 | 66009-1-Ig | Proteintech |
| Phospho-Rb (Ser807/811) | 1:1000 | 8516S | CST |
| LaminB1 | 1:1000 | 13435S | CST |
| p27 Kip1 | 1:1000 | 3686S | CST |
| p21 Waf1/Cip1 | 1:1000 | 2947S | CST |
| γH2AX | 1:500 | sc-517336 | Santa Cruz |
| TOP2A | 1:250 | sc-365916 | Santa Cruz |
| Ubiquitin | 1:1000 | 10201-2-AP | Proteintech |
| Parkin | 1:500 | 32833S | CST |
| MYC tag | 1:1000 | 60003-2-Ig | Proteintech |
| HA Tag | 1:1000 | 81290-1-RR | Proteintech |
| FOXM1 | 1:1000 | 20459S | CST |

**Supplementary Table S3. The sequences of primers used in the study.**

| Primer | Sequence |
| --- | --- |
| UBE2C-Forward | GACCTGAGGTATAAGCTCTCGC |
| UBE2C-Reverse | TTACCCTGGGTGTCCACGTT |
| TOP2A-Forward | ACCATTGCAGCCTGTAAATGA |
| TOP2A-Reverse | GGGCGGAGCAAAATATGTTCC |
| FOXM1-1-Forward | GAGCGTCTCATTGGCTGGAT |
| FOXM1-1-Reverse | GGCAGAGAGACAGGAACTCG |
| FOXM1-2-Forward | ACCCAAGCGAGCCATTGATT |
| FOXM1-2-Reverse | ACAGGAACTCGGAGAACACG |
| FOXM1-3-Forward | ATCAAACCCAAGCGAGCCAT |
| FOXM1-3-Reverse | CTCGGAGAACACGACTGCAA |
| FOXM1-4-Forward | CATTGGCTGGATCAAACCCA |
| FOXM1-4-Reverse | GGAGAACACGACTGCAACTG |
| GAPDH-Forward | GGAGCGAGATCCCTCCAAAAT |
| GAPDH-Reverse | GGCTGTTGTCATACTTCTCATGG |

**Supplementary figures**


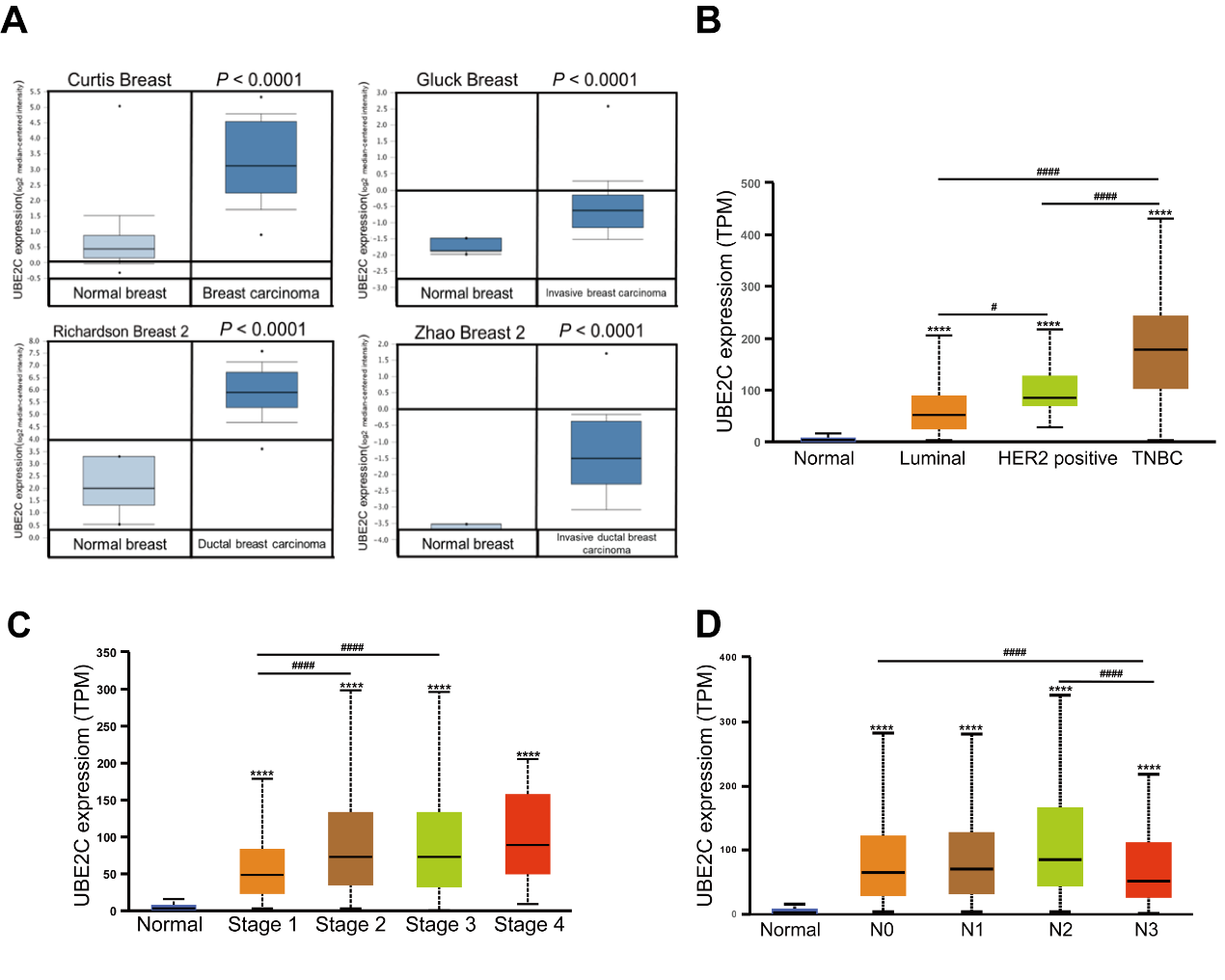


**Figure S1. UBE2C is overexpressed in breast cancer. A** Oncomine database was used to analyze the expression of UBE2C in breast cancer. **B** UALCAN database was used to analyze the expression of UBE2C in different subtypes of breast cancer. **C** UALCAN database was used to analyze the expression of UBE2C in different stages of breast cancer. **D** UALCAN database was used to analyze the expression of UBE2C in different histological grades of breast cancer. **P* < 0.05, ***P* < 0.01, ****P* < 0.001, *****P* < 0.0001 vs. normal tissue group.


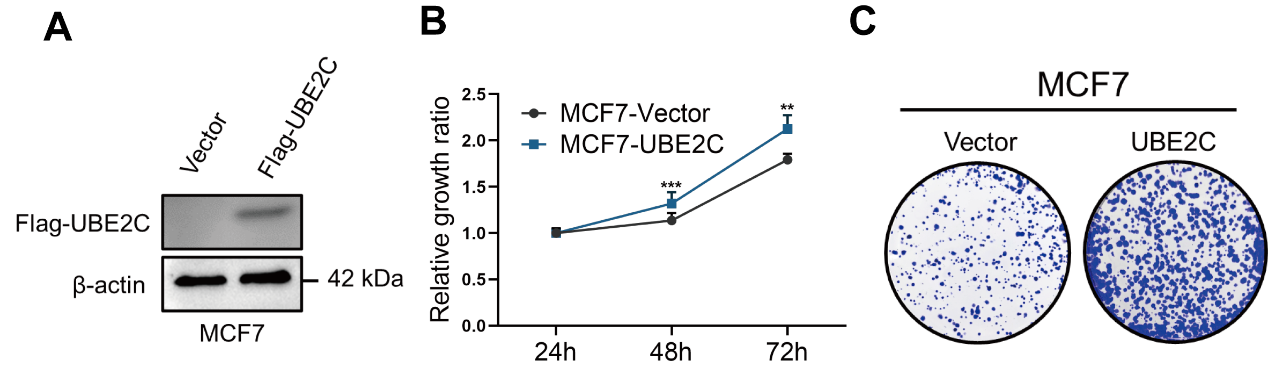


**Figure S2. Overexpression of UBE2C promotes the proliferation of breast cancer cells. A** Western blot was carried out to validate the overexpression of UBE2C in MCF7 cells. **B** CCK8 assay was carried out to verify the effect of UBE2C overexpression on the proliferation of MCF7 cells. **C** Colony formation assay was performed to verify the effect of UBE2C overexpression on the colony formation of MCF7 cells. **P* < 0.05, ***P* < 0.01, ****P* < 0.001, *****P* < 0.0001 vs. 24h group.


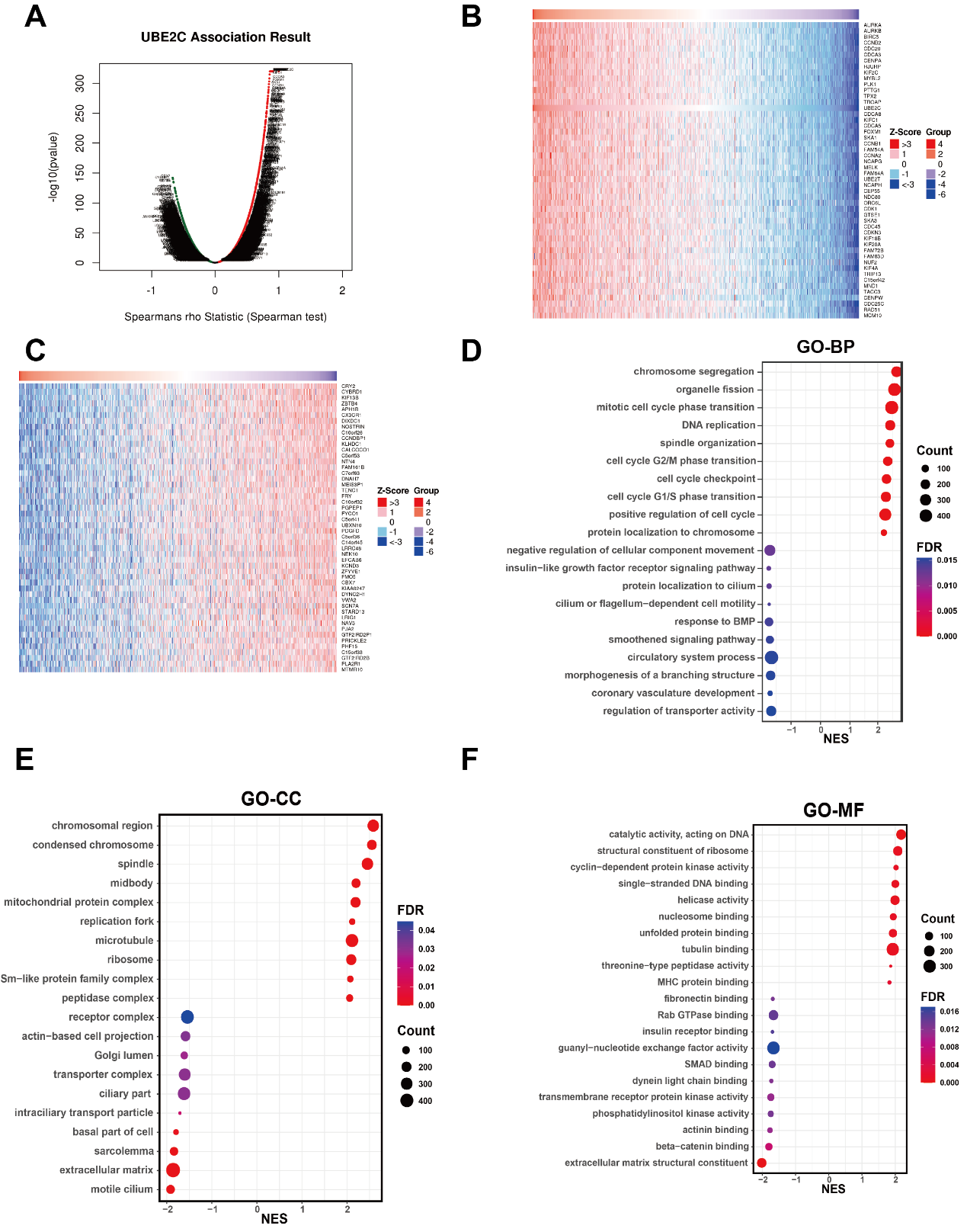


**Figure S3. Bioinformatics analysis of UBE2C associated genes based on TCGA breast cancer cohort. A** Genes that positively correlated with UBE2C and negatively correlated with UBE2C were plotted in the volcano plot. **B** TOP 50 genes positively correlated with UBE2C were depicted in heatmap. **C** TOP 50 genes negatively correlated with UBE2C were depicted in heatmap. **D** GO enrichment was performed in terms of biological process. **E** GO enrichment was performed in terms of cellular component. **F** GO enrichment was performed in terms of molecular function.


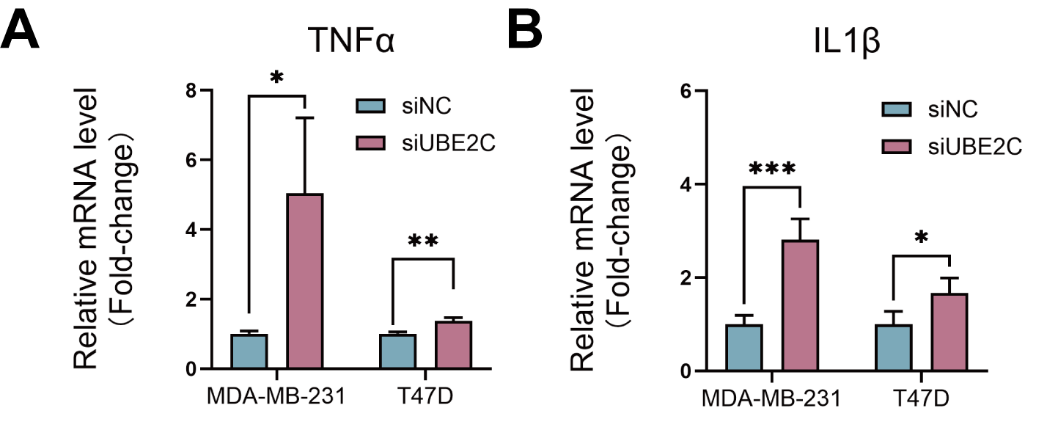


**Figure S4. UBE2C knockdown promoted the expression of SASP factors TNFα and IL1β. A** The mRNA level of TNFα after UBE2C knockdown was validated by RT-qPCR. B The mRNA level of IL1β after UBE2C knockdown was validated by RT-qPCR. (**P* < 0.05, ****P* < 0.001 vs. siNC group.)


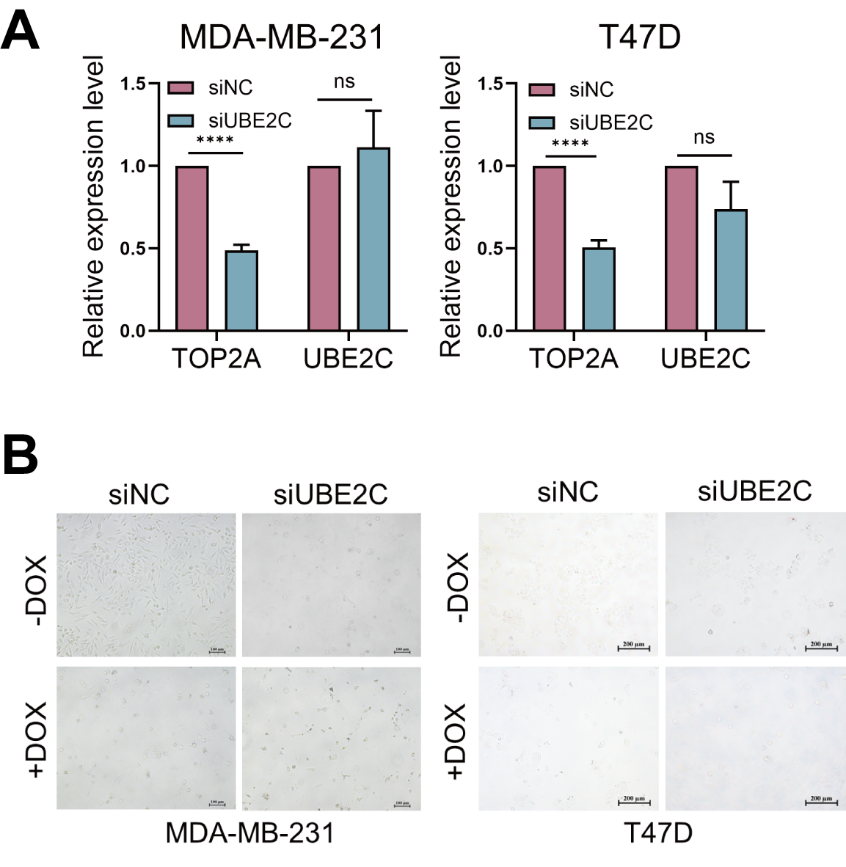


**Figure S5. Inhibition of UBE2C sensitizes breast cancer cells to doxorubicin in vitro. A** The quantification result of Western blot in Fig.3A. **B** The representative images of cell morphology was captured after MDA-MB-231 and T47D being treated with doxorubicin when UBE2C was knocked down. (****P* < 0.001, *****P* < 0.0001 vs. siNC group.)


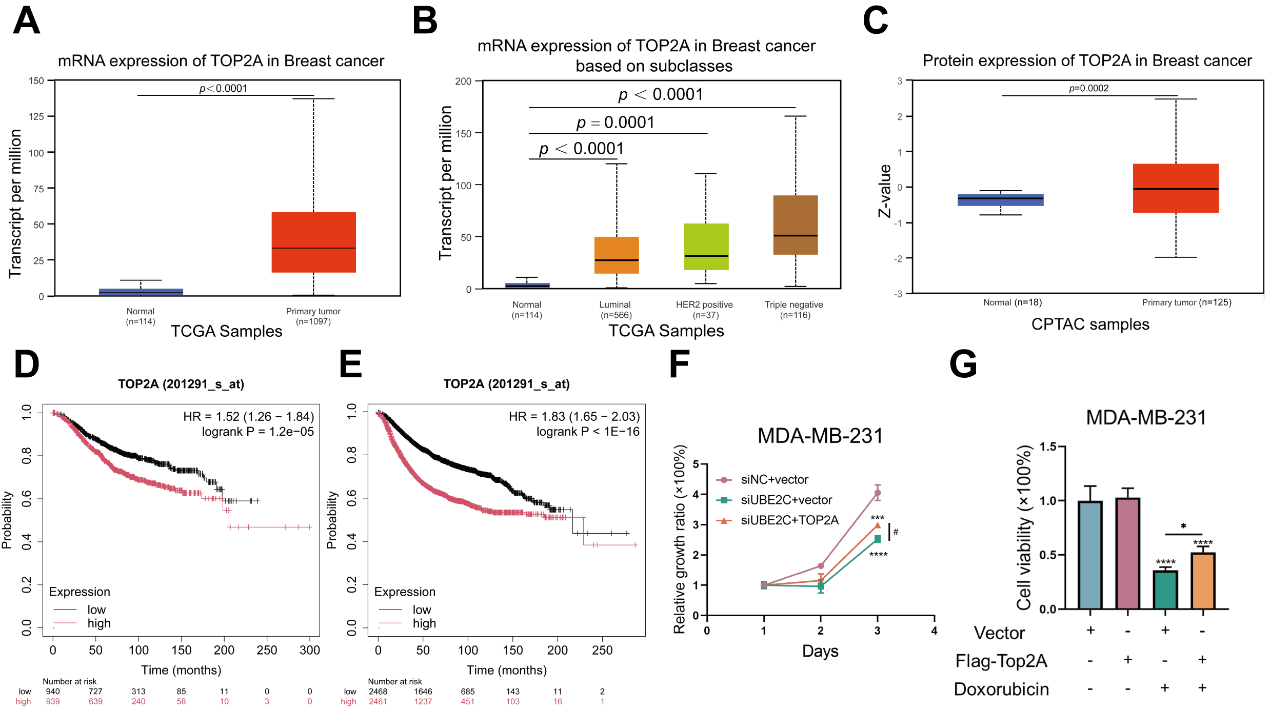


**Figure S6. TOP2A is upregulated in breast cancer and correlated with poor prognosis of breast cancer patients. A** UALCAN database was used to analyze the mRNA level of TOP2A in breast cancer patients based on TCGA cohorts. **B** UALCAN database was used to analyze the mRNA level of TOP2A in different subtypes of breast cancer patients based on TCGA cohorts. **C** UALCAN database was used to analyze the protein level of TOP2A in breast cancer patients based on TCGA cohorts. **D** KMPlotter database was used to analyze the correlation of the expression of TOP2A with the overall survival of breast cancer patients. **E** KMPlotter database was used to analyze the correlation of the expression of TOP2A with the relapse free survival (RFS) of breast cancer patients. **F** CCK8 assay was performed to verify the effect of overexpression of TOP2A on the cell growth inhibition caused by UBE2C knockdown in MDA-MB-231 cells. **G** CCK8 assay was performed to verify the effect of overexpression of TOP2A on the cell growth inhibition caused by doxorubicin treatment in MDA-MB-231 cells. ****P* < 0.001, *****P* < 0.0001 vs. siNC+Vector group. *^#^P* < 0.05 between two groups.


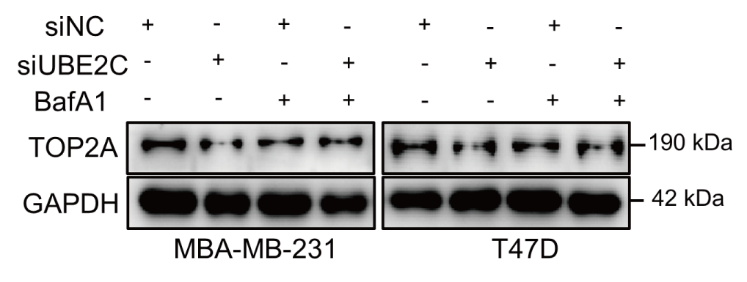


**Figure S7. The effect of BafA1 on the expression of TOP2A after UBE2C knockdown.**
